# Supplementary figures and images for: A novel heteromeric pantothenate kinase complex in apicomplexan parasites
Source: PLoS Pathog. 2021 Jul 29;17(7):e1009797. doi: 10.1371/journal.ppat.1009797 (PMC8366970; doi:10.1371/journal.ppat.1009797)

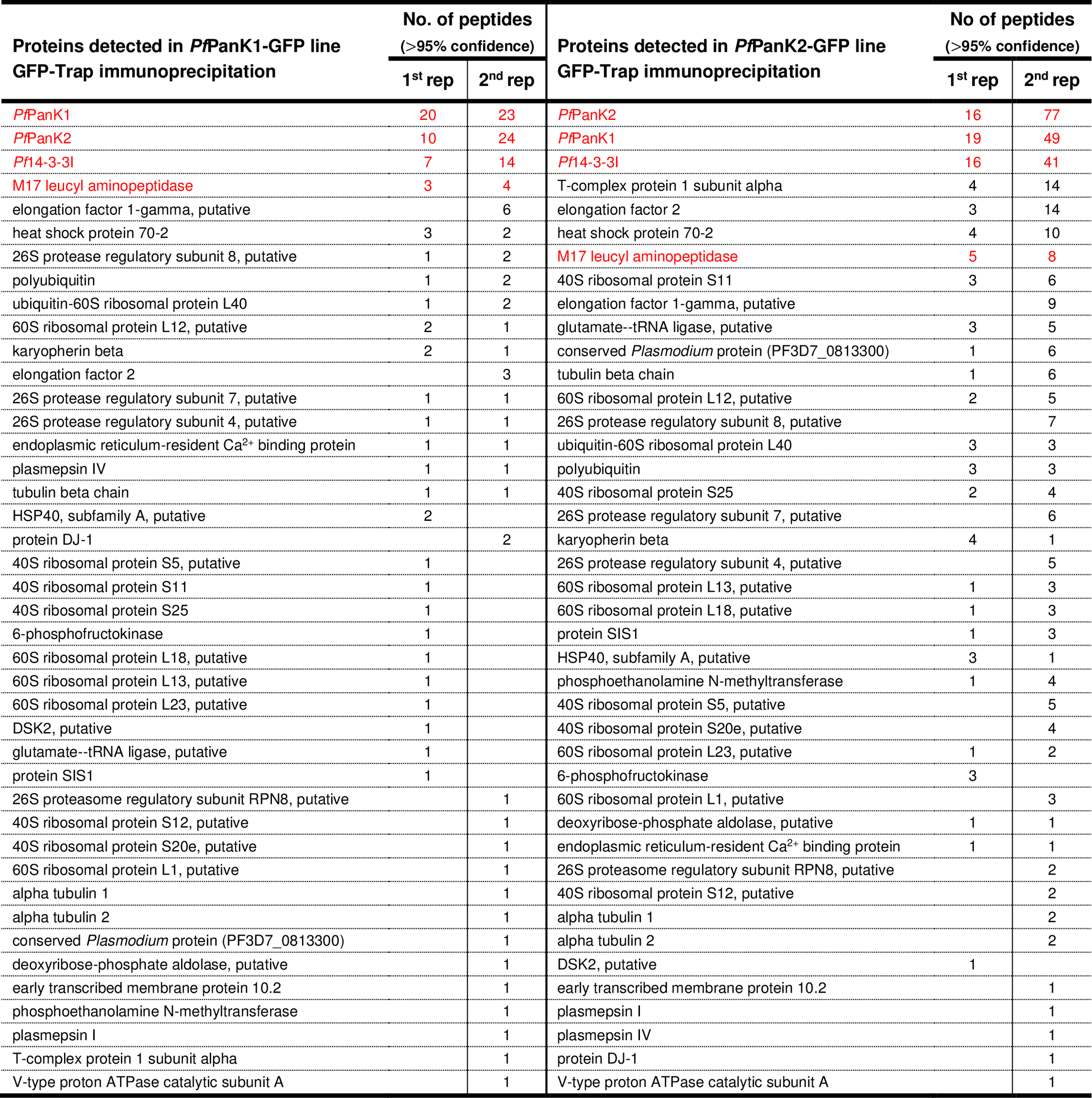

Supplement: S1 Table — Proteins detected in each immunoprecipitation experiment are listed in descending order according of the total number of peptides detected across the two replicates (total peptides in 1st and 2nd rep columns). Only proteins that are present in the immunoprecipitation fractions of both parasite lines and absent in the negative controls (bound fractions of untagged GFP and 3D7 parasite lysates) are shown. Proteins shown in Fig 2A are indicated in red. (TIF) [file ppat.1009797.s001.tif]

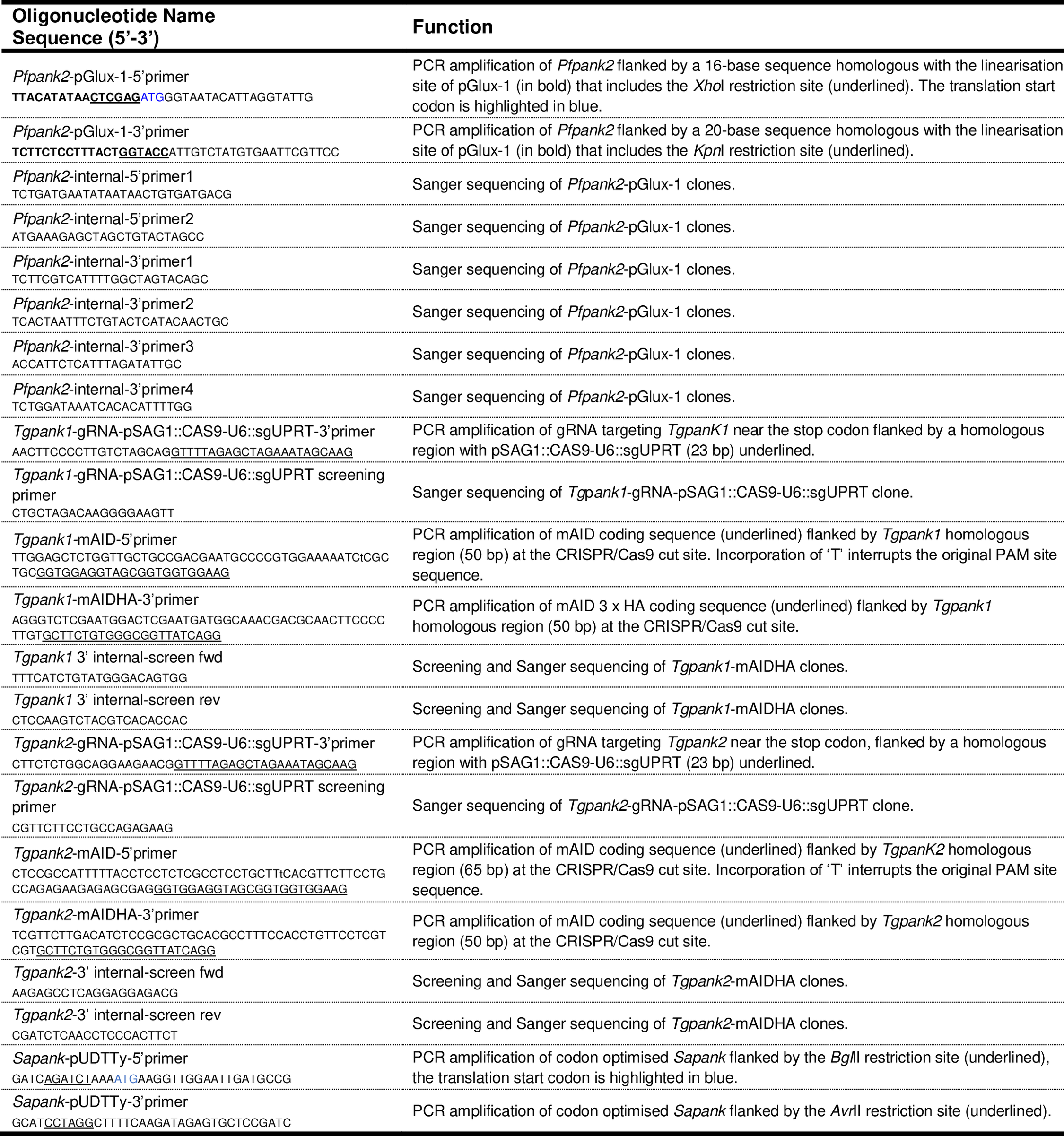

Supplement: S2 Table — (TIF) [file ppat.1009797.s002.tif]

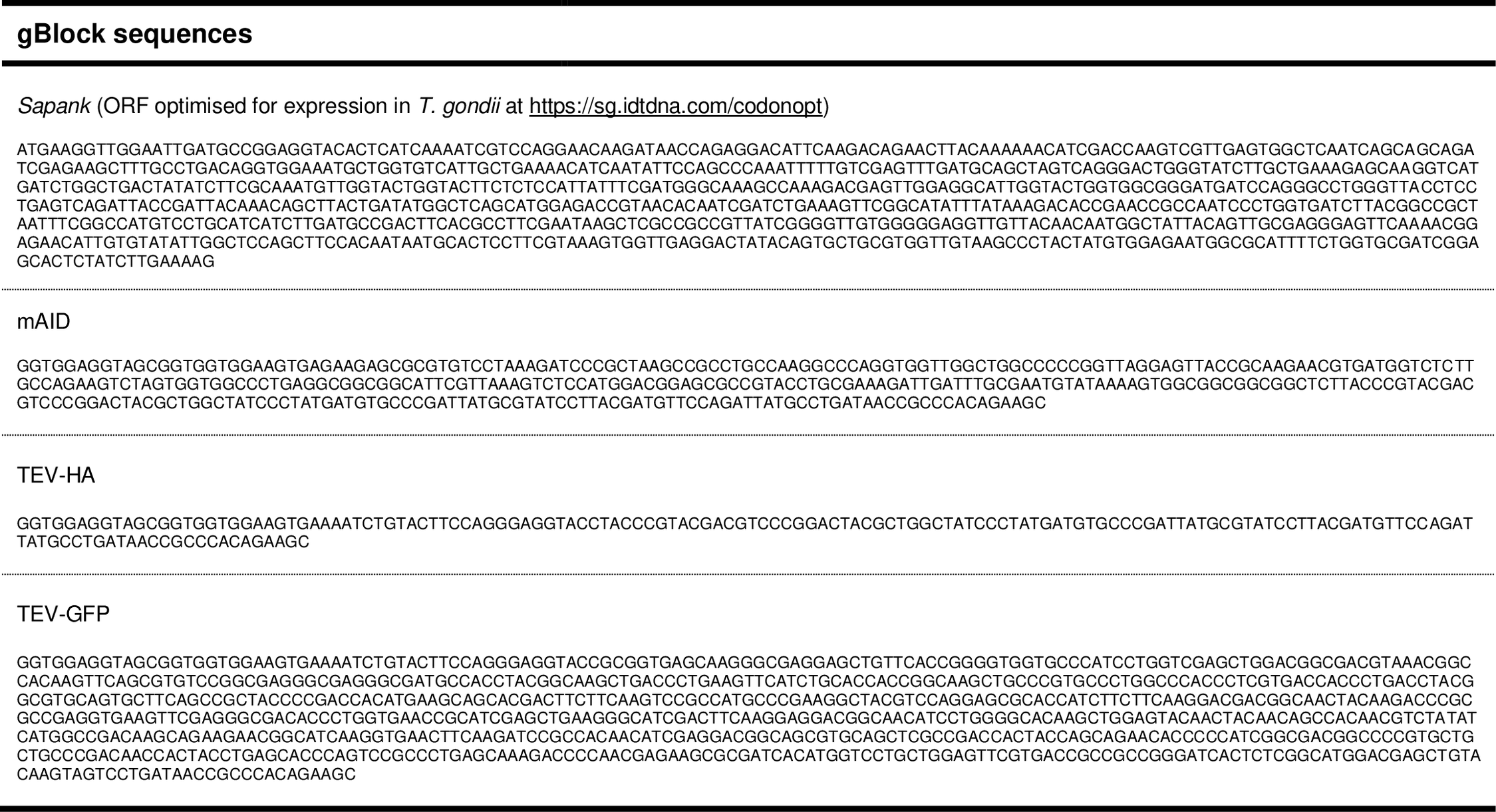

Supplement: S3 Table — (TIF) [file ppat.1009797.s003.tif]

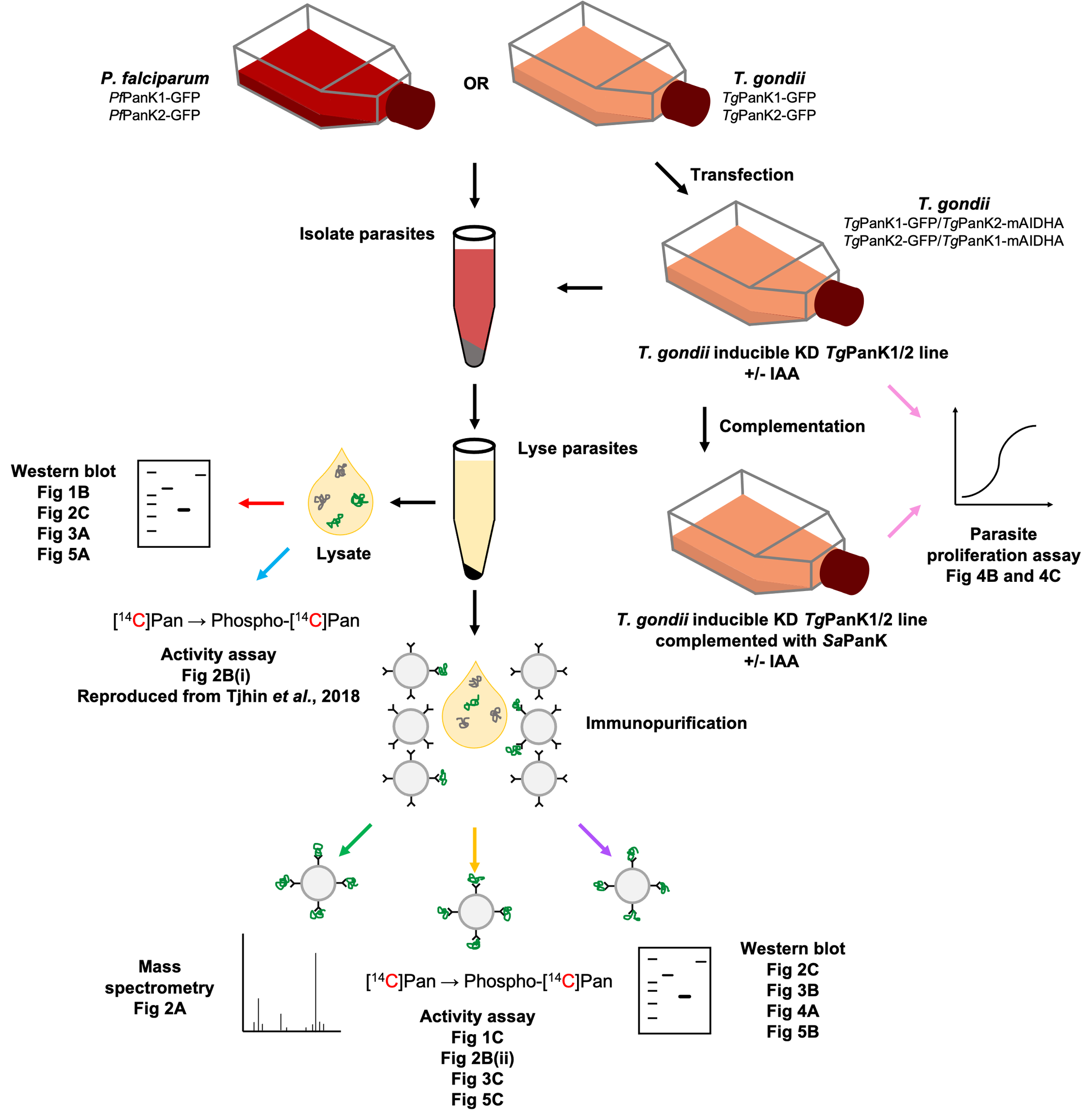

Supplement: S1 Fig — Flow chart highlighting the cell lines generated for the study and the main experimental steps that were performed. The coloured arrows represent final experimental results, and the associated figures within which the data are presented, are indicated under each experiment. (TIF) [file ppat.1009797.s004.tif]

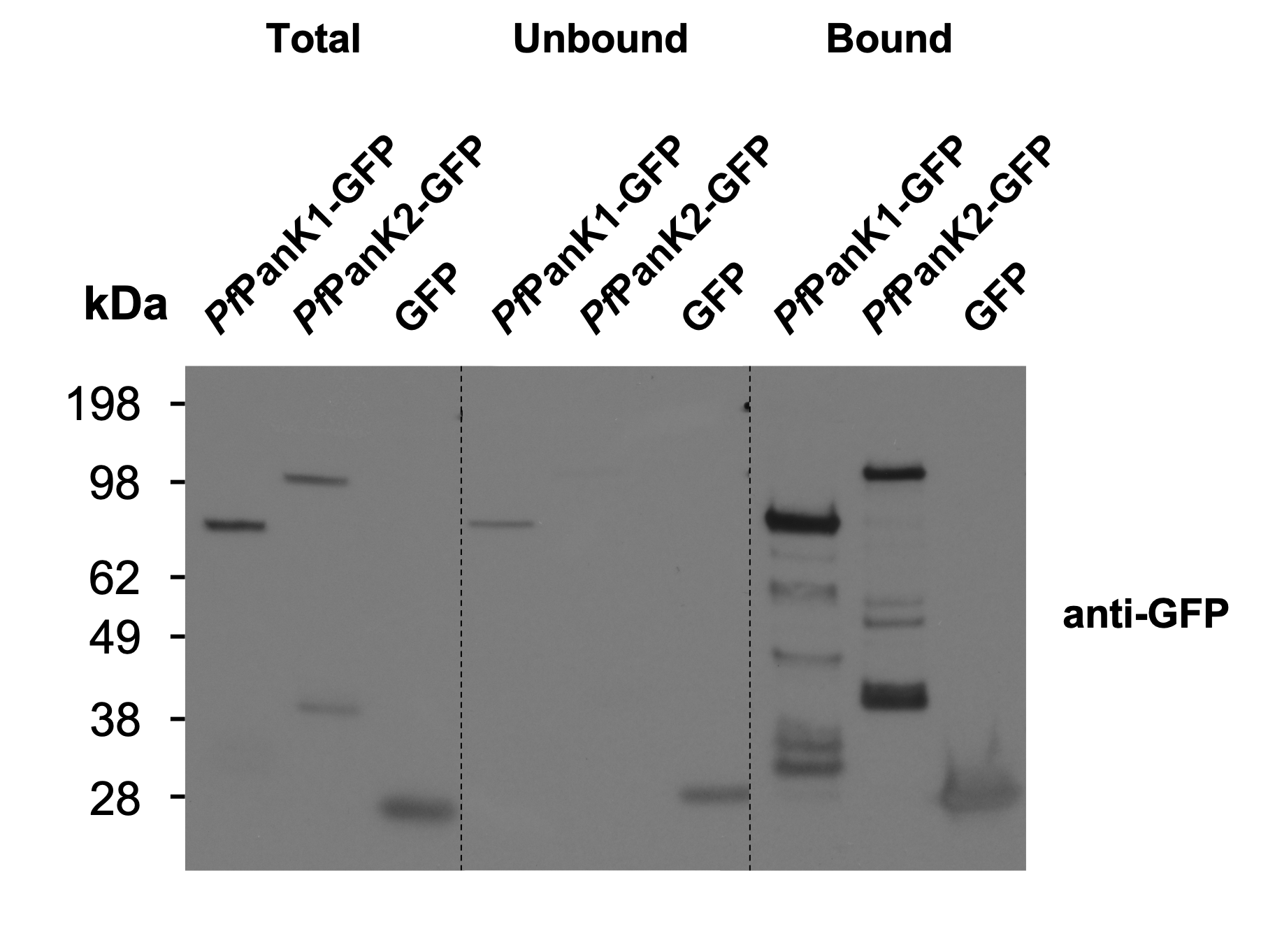

Supplement: S2 Fig — Denaturing western blot analysis of the GFP-tagged proteins present in the total lysate, unbound and GFP-Trap-bound fractions of PfPanK1-GFP, PfPanK2-GFP and untagged GFP lines. Western blots were performed with anti-GFP antibody and the blot shown is representative of two independent experiments each performed with a different batch of parasites. (TIF) [file ppat.1009797.s005.tif]

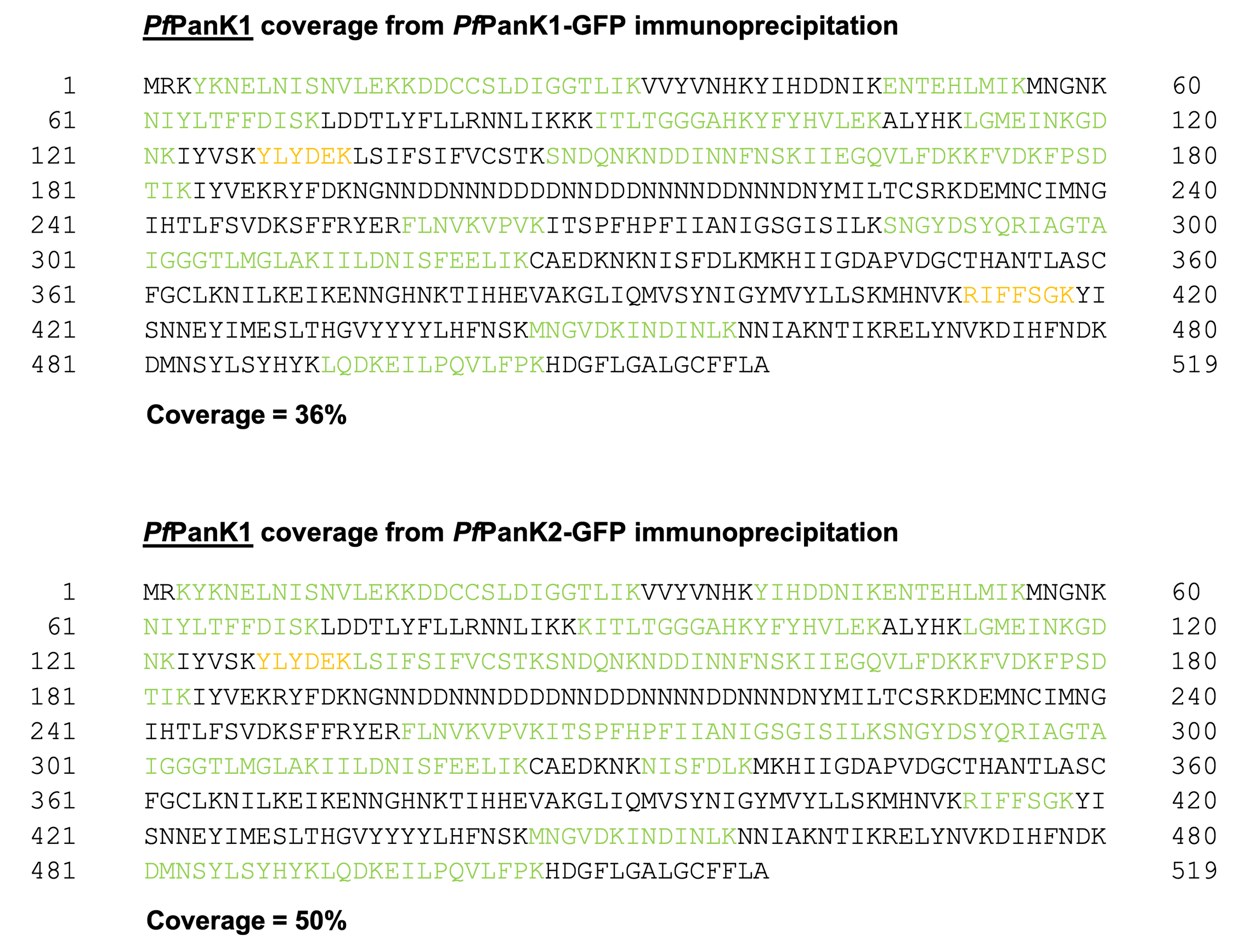

Supplement: S3 Fig — PfPanK1 peptides detected in the two independent MS analyses of the GFP-Trap immunoprecipitation from the PfPanK1-GFP and PfPanK2-GFP lines. Residues in green were detected in either analysis with >95% confidence, while residues in orange were detected in either analysis with >90% (but <95%) confidence. Percentage coverage was calculated using only the residues labelled green. (TIF) [file ppat.1009797.s006.tif]

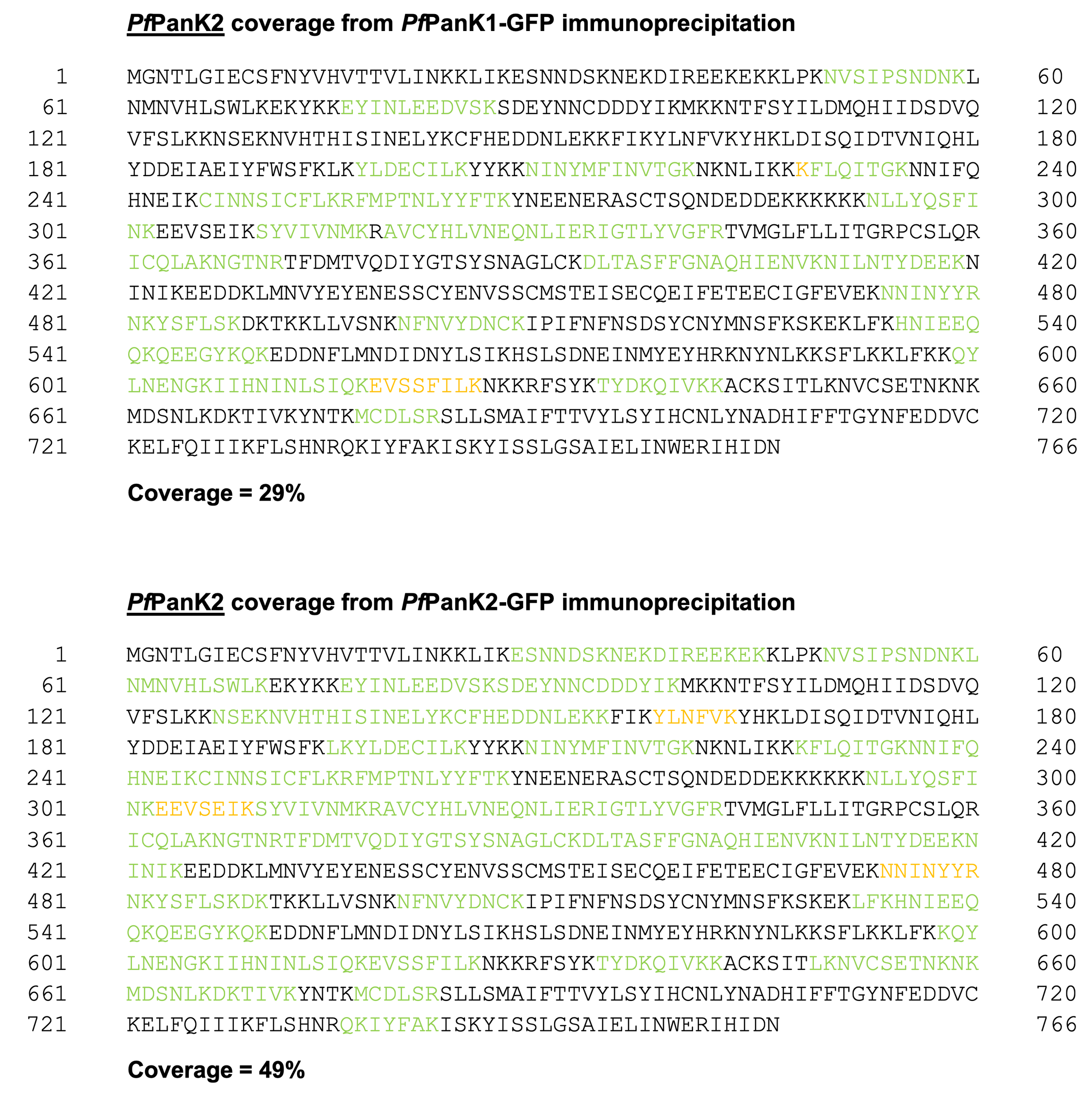

Supplement: S4 Fig — PfPanK2 peptides detected in the two independent MS analyses of the GFP-Trap immunoprecipitation from the PfPanK1-GFP and PfPanK2-GFP lines. Residues in green were detected in either analysis with >95% confidence, while residues in orange were detected in either analysis with >90% (but <95%) confidence. Percentage coverage was calculated using only the residues labelled green. (TIF) [file ppat.1009797.s007.tif]

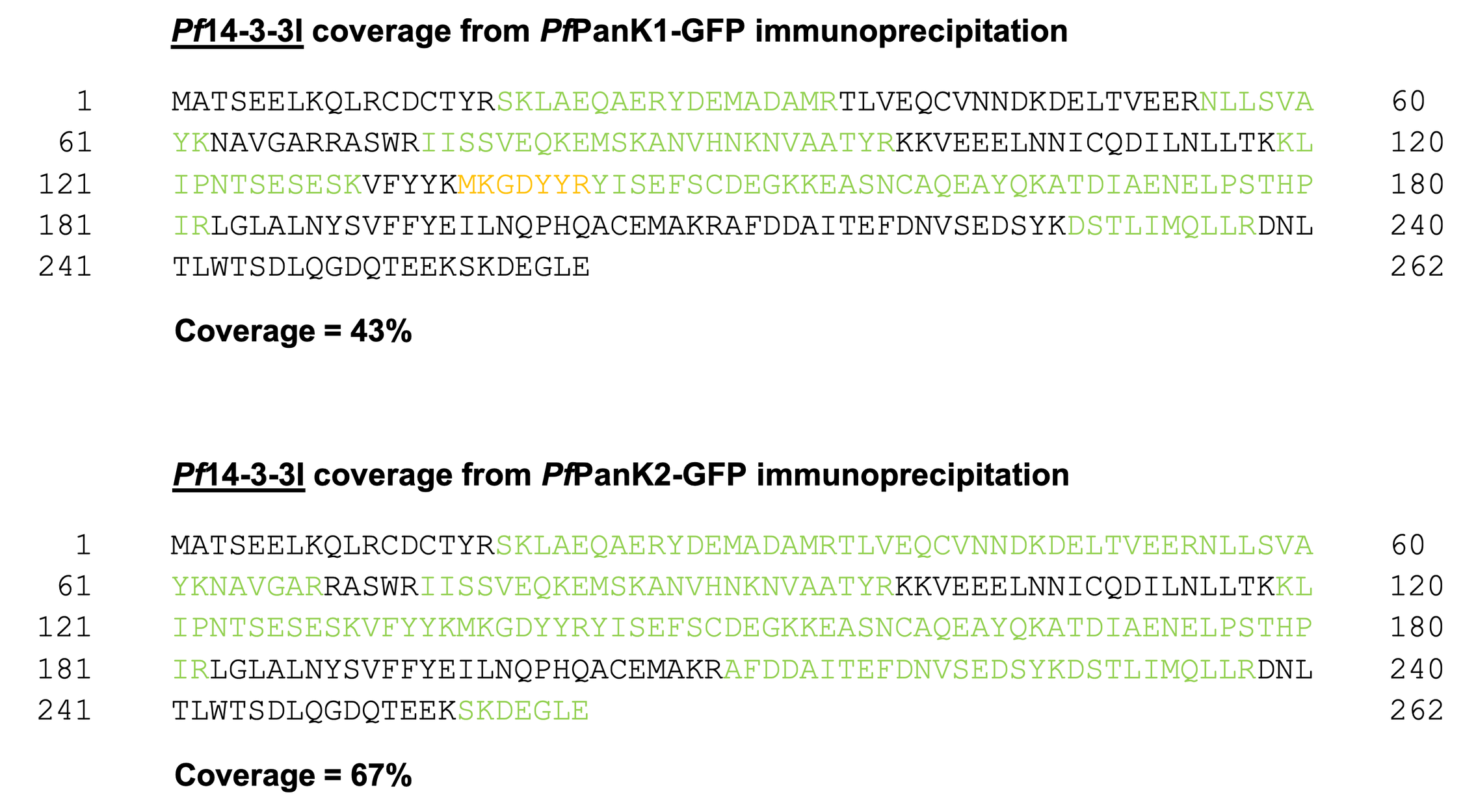

Supplement: S5 Fig — Pf14-3-3I peptides detected in the two independent MS analyses of the GFP-Trap immunoprecipitation from the PfPanK1-GFP and PfPanK2-GFP lines. Residues in green were detected in either analysis with >95% confidence, while residues in orange were detected in either analysis with >90% (but <95%) confidence. Percentage coverage was calculated using only the residues labelled green. (TIF) [file ppat.1009797.s008.tif]

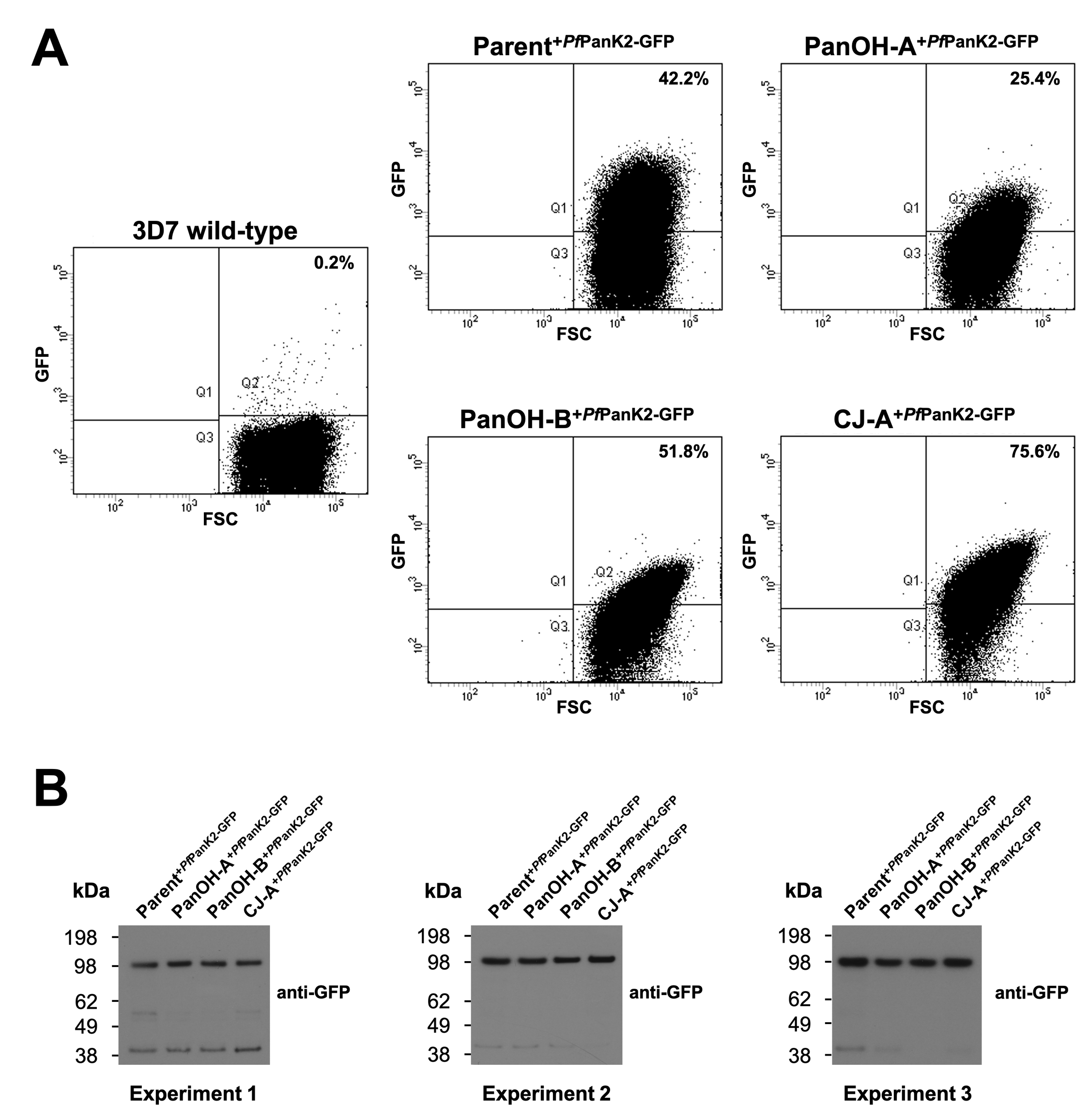

Supplement: S6 Fig — (A) The proportion of GFP-positive saponin-isolated 3D7, Parent+PfPanK2-GFP, PanOH-A+PfPanK2-GFP, PanOH-B+PfPanK2-GFP and CJ-A+PfPanK2-GFP trophozoites was determined by FACS analysis. The forward scatter (FSC) intensity on each x-axis corresponds to cell size and the y-axis corresponds to the intensity of GFP fluorescence. The proportion of GFP-positive cells in each transgenic line (percentage value in each plot) was determined by using 3D7 trophozoites to set a gating threshold below which parasites were defined to be auto-fluorescent. Data shown are representative of three independent experiments, each performed prior to the [14C]pantothenate phosphorylation assays presented in Fig 2B(ii). The flow cytometry data were used to standardise the amount of PfPanK2-GFP immunoprecipitated from each cell line used in each [14C]pantothenate phosphorylation assay. (B) Denaturing western blot analysis of PfPanK2-GFP in the GFP-Trap immunoprecipitated complexes that were used in the [14C]pantothenate phosphorylation assays performed to generate the data in Fig 2B(ii). Western blots were performed with an anti-GFP antibody and each blot shows the relative amounts of PfPanK2-GFP immunopurified from the four different cell lines used in each of the three [14C]pantothenate phosphorylation experiment. The same volume of samples (10 μL per lane) was used for all three experiments. (TIF) [file ppat.1009797.s009.tif]

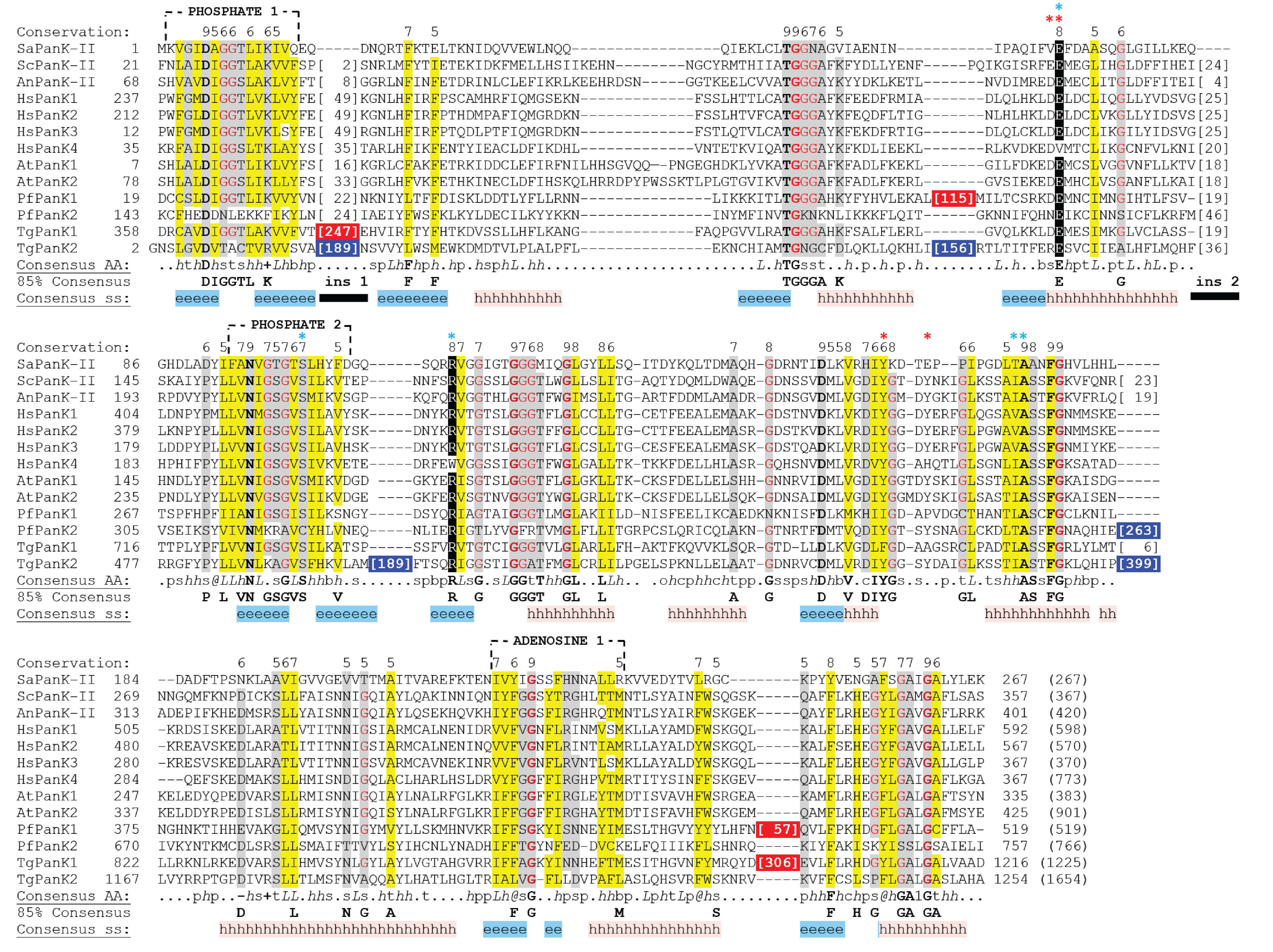

Supplement: S7 Fig — The conserved PHOSPHATE 1, PHOSPHATE 2, and ADENOSINE 1 motifs of the acetate and sugar kinases/Hsc70/actin (ASKHA) superfamily of kinases are labelled at the top of the alignment. The Glu (E) residue involved in catalysis and the Arg (R) residue involved in positioning the substrate, are shown on a black background. Residues that have been found to interact with pantothenate and acetyl-CoA in human PanK3 [34,35] are marked with a blue asterisk. Residues that were found to interact to stabilise the human PanK3 active site are marked with a red asterisk. The catalytic Glu (E) residue is marked with a red and blue asterisk as it is involved in both the interaction with pantothenate and the stabilisation of the active site through interaction with a Tyr (Y) residue of the opposite protomer. The numbers at the start and end of each sequence indicate the position of the first and last residue in the alignment, respectively. The lengths of insertions are specified within the square brackets and the total length of protein sequences are shown in round brackets. Residues within the ASKHA superfamily motifs and conserved residues are highlighted based on the consensus AA guide for the column as follows: identical = bold, hydrophobic (W,F,Y,M,L,I,V,A,C,T,H) = yellow, charged/polar/small (D,E,K,R,H/D,E,H,K,N,Q,R,S,T/A,G,C,S,V,N,D,T,P) = grey and Gly (G) = red. The two insertion regions (Ins 1 and Ins 2) common to eukaryotic type II PanKs, but absent in prokaryotic PanKs are indicated by the black horizontal bars, while the PfPank1/TgPanK1 and PfPanK2/TgPanK2 specific inserts are highlighted on a red and blue background, respectively. Conservation refers to the conservation index [77]. Values at and above the conservation index cut-off (5) are displayed above the amino acid. Consensus AA: refers to the consensus level alignment parameters for the consensus amino acid sequence. This is displayed if the weighted frequency of a certain class of residues in a position is above 0.8. [file ppat.1009797.s010.tif]

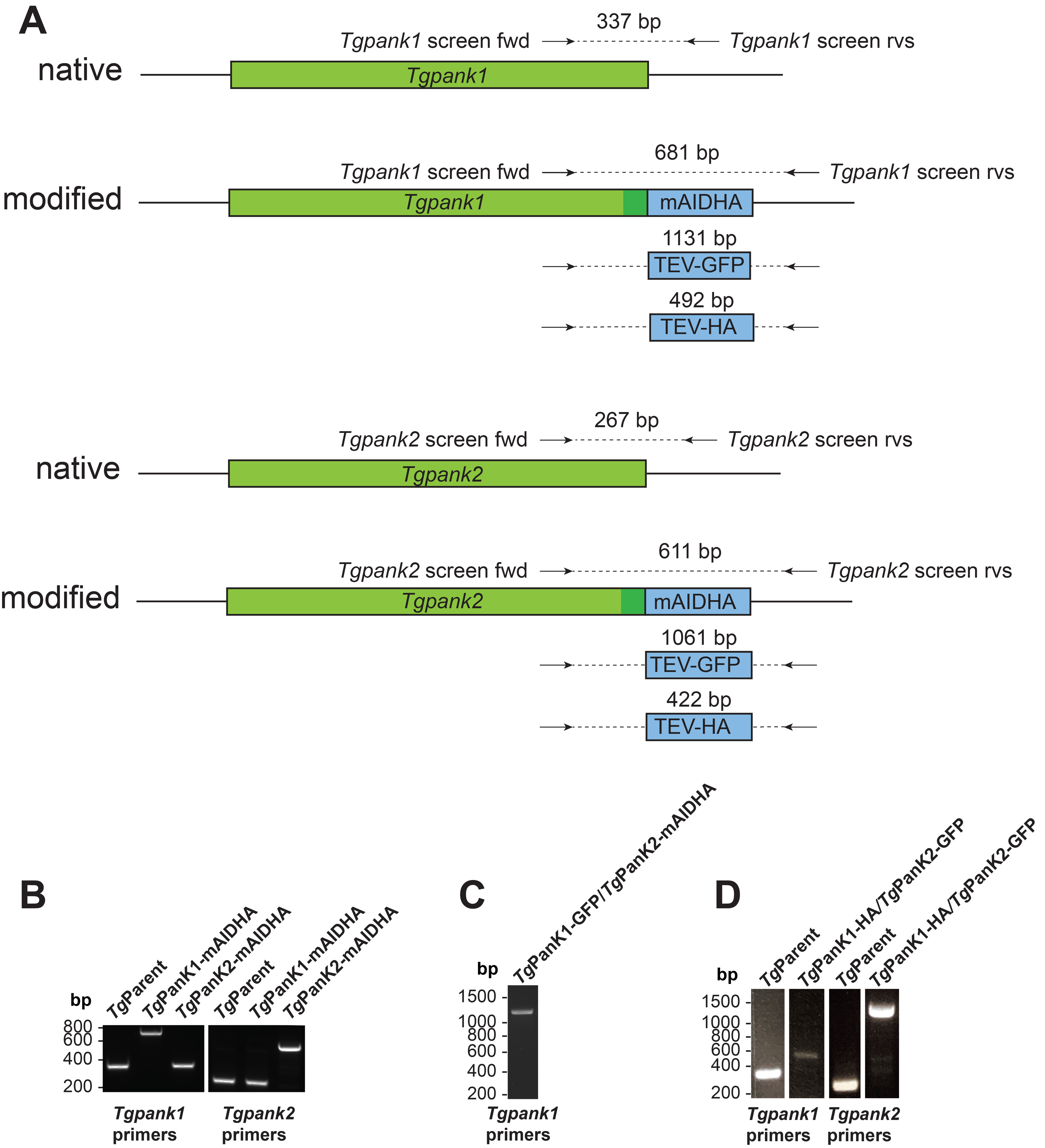

Supplement: S8 Fig — (A) Schematic of theTgpank1 and Tgpank2 genomic loci, indicating the incorporation sites of the epitope tag coding sequence. The expected sizes of the PCR products when screened with each set of screening primers are shown above the corresponding epitope tag coding sequence. The screening primers are Tgpank1 screen fwd and rvs for Tgpank1 (referred to as Tgpank1 primers in panels B-D), and Tgpank2 screen fwd and rvs for Tgpank2 (referred to as Tgpank2 primers in panels B-D). Primers are detailed in S2 Table. (B) PCR analysis of the parental strain (TgParent), and singly-tagged TgPanK1-mAIDHA and TgPanK2-mAIDHA lines. Both TgPanK1-mAIDHA and TgPanK2-mAIDHA have successfully incorporated mAIDHA tags. (C) PCR analysis of the doubly-tagged TgPanK1-GFP/TgPanK2-mAIDHA line. CRISPR/Cas9 was utilised to incorporate a sequence encoding a TEV-GFP tag into the genomic locus of the Tgpank1 gene within the TgPanK2-mAIDHA line. (D) PCR analysis of the TgPanK1-HA/TgPanK2-GFP doubly-tagged line. CRISPR/Cas9 was utilised to incorporate a sequence encoding a TEV-HA tag into the genomic locus of the Tgpank1 gene and a sequence encoding a TEV-GFP tag into the genomic locus of the Tgpank2 gene. (TIF) [file ppat.1009797.s011.tif]

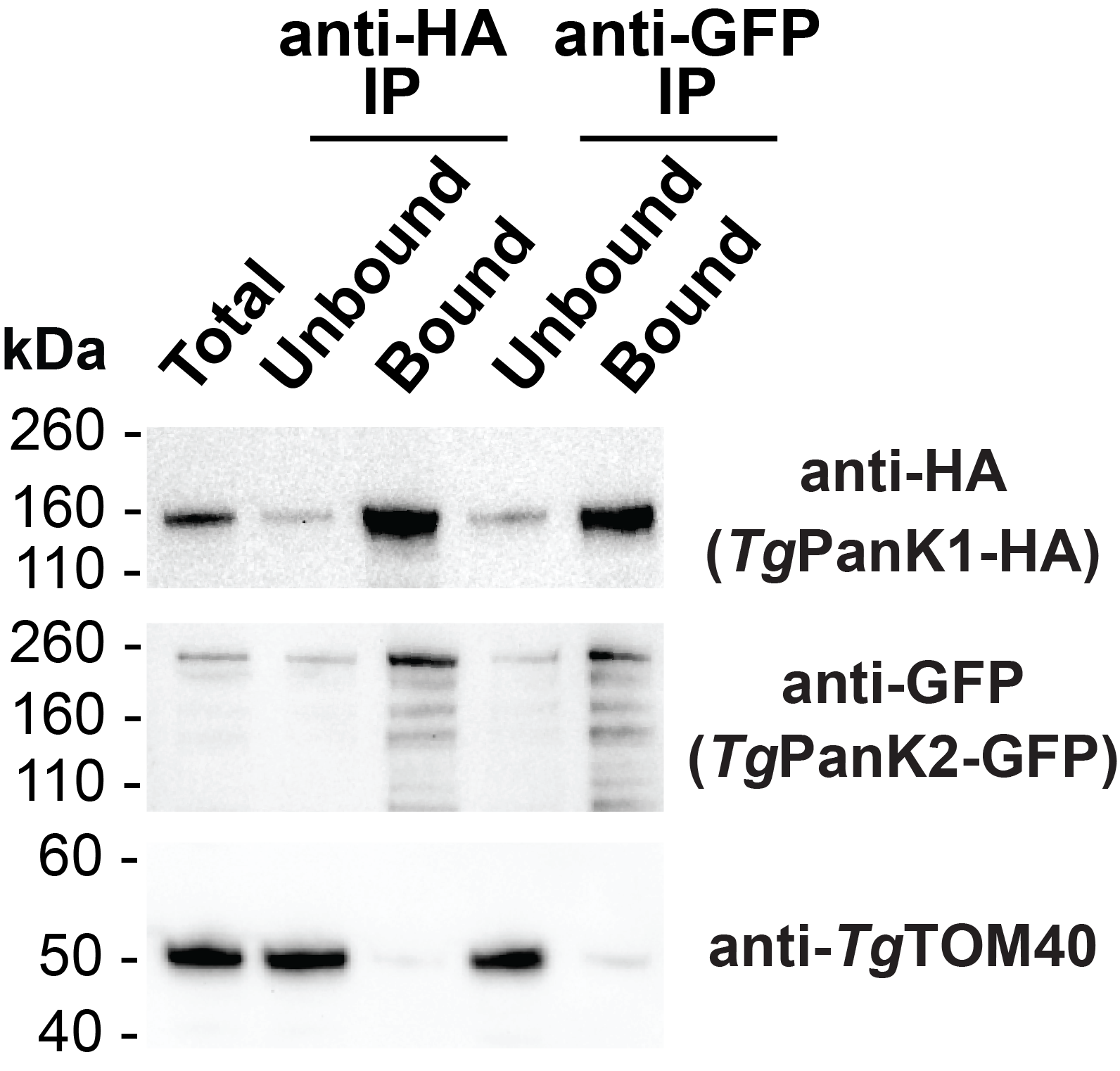

Supplement: S9 Fig — Anti-HA and anti-GFP denaturing western blot analysis of fractions from GFP-Trap and anti-HA immunoprecipitations performed using lysates prepared from the parasite lines expressing TgPanK1-HA/TgPanK2-GFP. The expected molecular masses of TgPanK1-HA and TgPanK2-GFP are 136 kDa and 206 kDa, respectively. The blot shown is representative of three independent experiments, each performed with a different batch of parasites. Denaturing western blots were also probed with anti-TgTOM40, which served as a control for a protein that is not part of the PanK complex. (TIF) [file ppat.1009797.s012.tif]

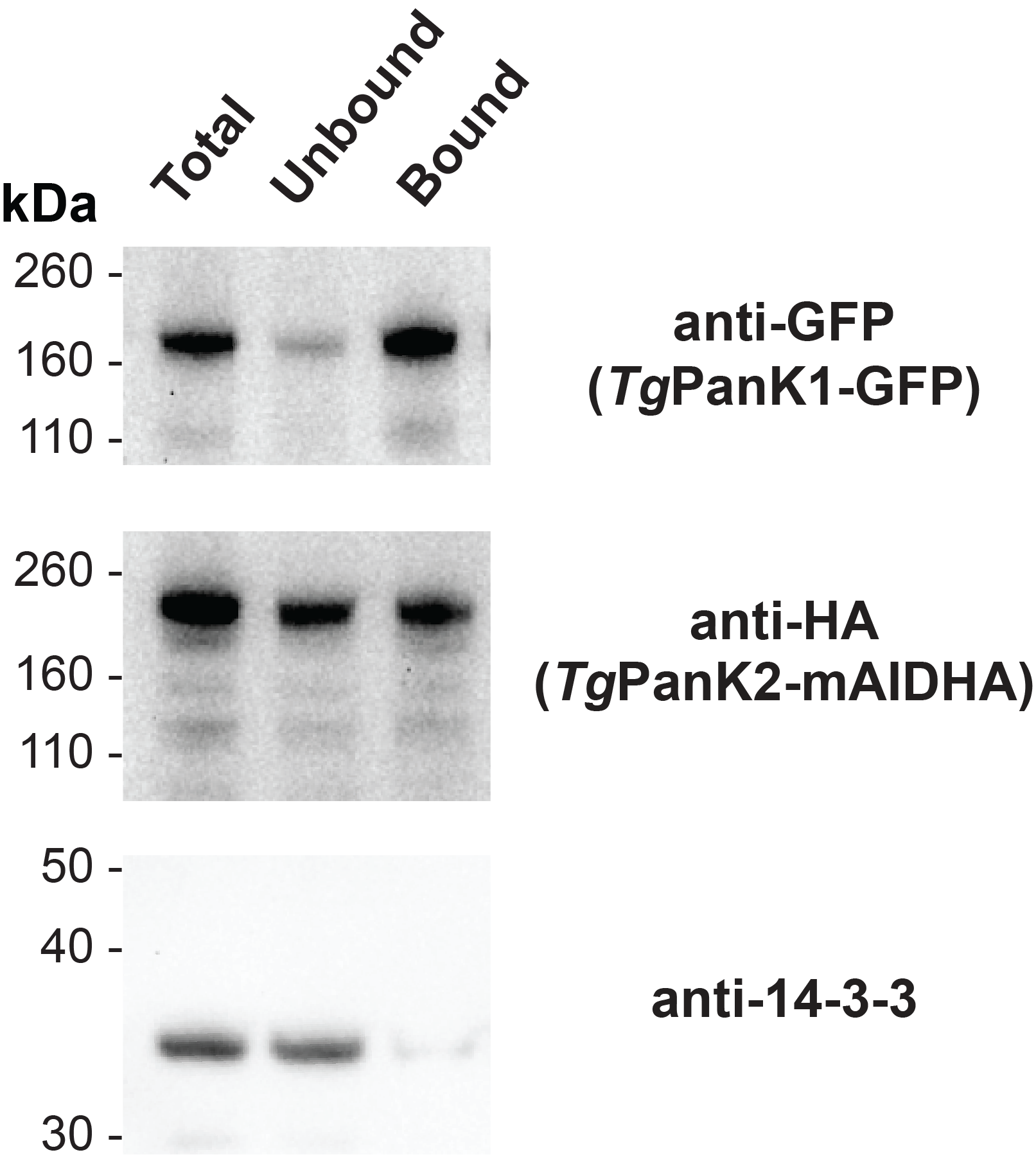

Supplement: S10 Fig — Anti-HA, anti-GFP and anti-14-3-3 denaturing western blot analysis of fractions from GFP-Trap immunoprecipitation of lysates prepared from the parasite line expressing TgPanK1-GFP/TgPanK2-mAIDHA. The expected molecular masses of TgPanK1-GFP, TgPanK2-mAIDHA and 14-3-3 are approximately 160 kDa, 189 kDa and 37 kDa, respectively. The blots shown are representative of two independent experiments, each performed with a different batch of parasites. (TIF) [file ppat.1009797.s013.tif]

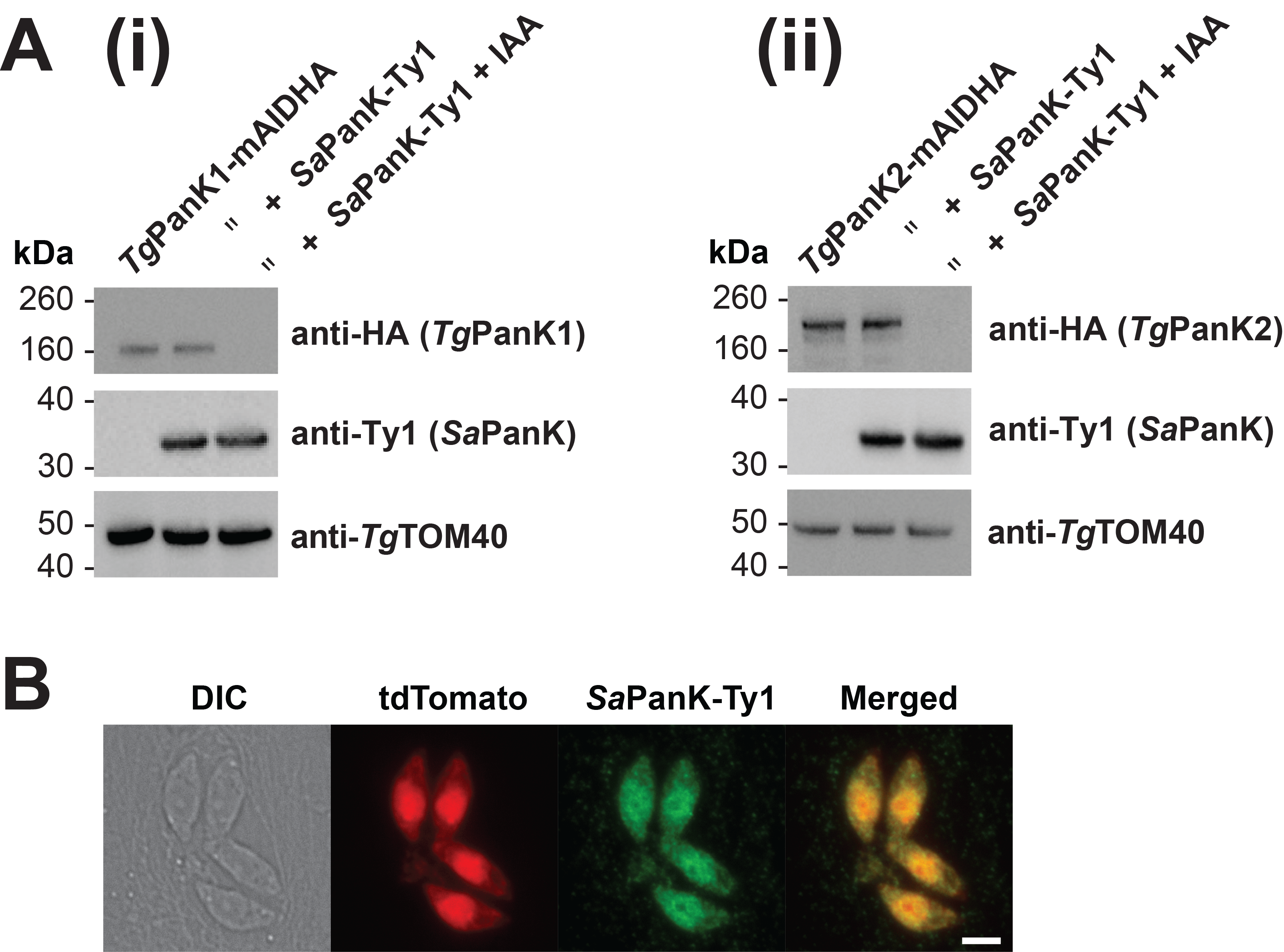

Supplement: S11 Fig — (A) Anti-HA and anti-Ty1 denaturing western blot analysis of SaPanK-Ty1-complemented and non-complemented (i) TgPanK1-mAIDHA and (ii) TgPanK2-mAIDHA lines, in the absence or presence (for 1 h) of 100 μM IAA. The expected molecular masses of TgPanK1-mAIDHA, TgPanK2-mAIDHA and SaPanK-Ty1 are ~143 kDa, ~189 kDa and ~29 kDa, respectively. Denaturing western blots were also probed with anti-TgTOM40, which served as a loading control. Each blot shown is representative of three independent experiments, each performed with a different batch of parasites. (B) Fluorescence micrographs of a HFF cell infected with four tachyzoite-stage TgPanK1-mAIDHA+SaPanK-Ty1 parasites within a vacuole, indicating the presence of SaPanK-Ty1. From left to right: Differential interference contrast (DIC), tdTomato RFP (a marker of the nucleus and cytosol; red), anti SaPanK-Ty1 (green), and merged images. Scale bar represents 2 μm. (TIF) [file ppat.1009797.s014.tif]

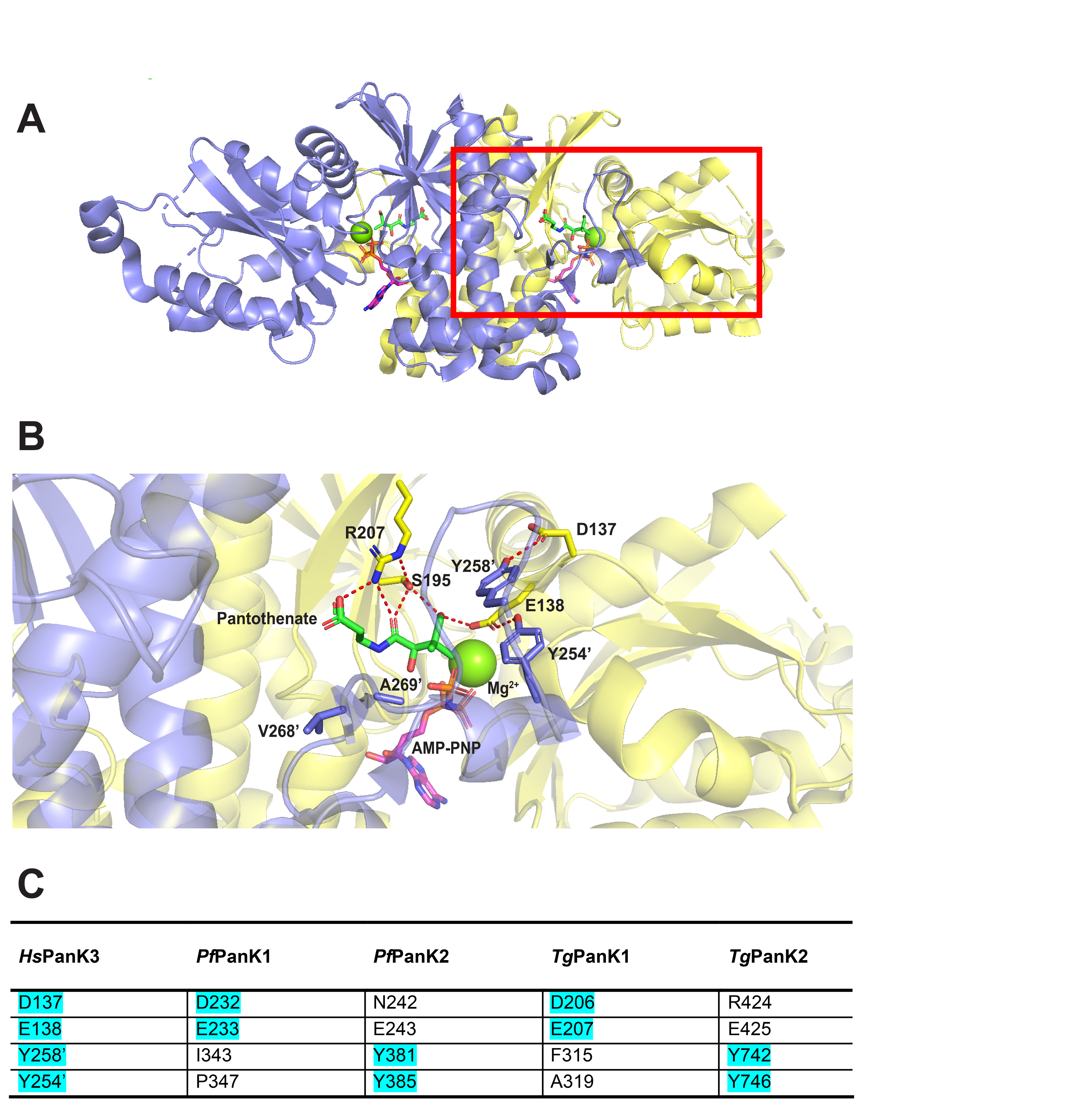

Supplement: S12 Fig — (A) H. sapiens AMP-PNP-pantothenate-bound PanK3 crystal structure (PDB ID: 5KPR, Subramanian et al. [35]). The homodimeric protein is made up of two identical protomers (lilac and yellow) forming two identical active sites, each binding pantothenate (carbon atoms shown in green). The red square encompasses one of the active sites. (B) Magnification of the region outlined by the red square in (A). Residues from both protomers contribute to the stabilisation of the binding pocket (E138 forms a hydrogen bond with Y254’ and D137 with Y258’) and interact with pantothenate (E138, S195, R207, A269’ and V268’). Hydrogen bonds with and between the sidechains of these residues are shown in red. An apostrophe denotes residues from the lilac protomer. (C) List of residues annotated in the HsPanK3 model that participate in the stabilisation of the binding pocket (highlighted cyan), and a comparison to the equivalent residues in P. falciparum and T. gondii PanKs. The PanKs from P. falciparum and T. gondii do not individually contain the complete set of residues required for the stabilisation of the binding pocket, but the combination of residues (highlighted cyan) from PanK1 and PanK2 suggests that each PanK1/PanK2 heterodimer will have only one stabilised binding site. (TIF) [file ppat.1009797.s015.tif]

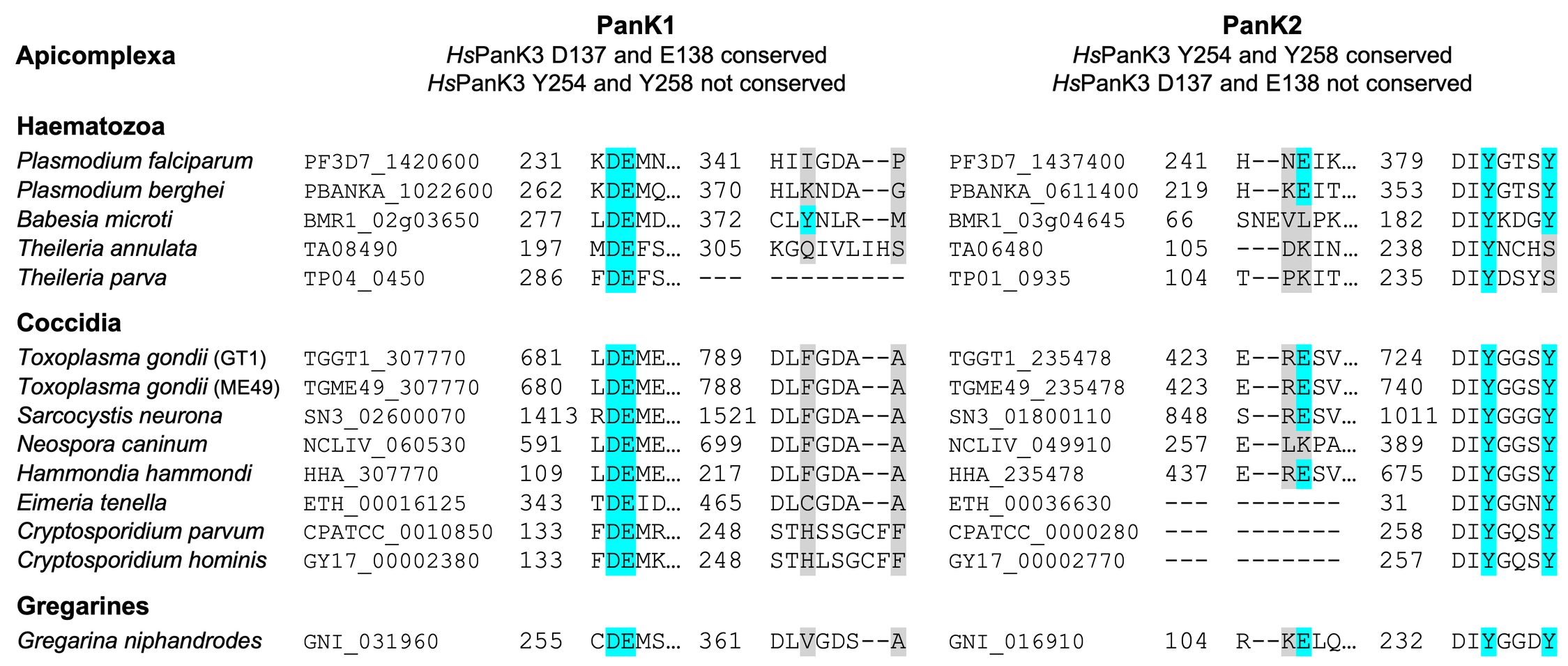

Supplement: S13 Fig — The apicomplexan PanK residues corresponding to the HsPanK3 residues that are involved in the stabilisation of the binding pocket (D137, E138, Y254 and Y258) are highlighted in cyan if they are conserved and in grey if they are not conserved. The numbers before each alignment indicate the position of the first residue in the alignment. Each apicomplexan PanK is grouped into PanK1 or PanK2 based on their similarity to either PfPanK1/TgPanK1 or PfPanK2/TgPanK2, respectively. The alignment was created using PROMALS3D [76]. (TIF) [file ppat.1009797.s016.tif]
