# Supplementary material for: A novel heteromeric pantothenate kinase complex in apicomplexan parasites
Source: PLoS Pathog. 2021 Jul 29;17(7):e1009797. doi: 10.1371/journal.ppat.1009797 (PMC8366970; doi:10.1371/journal.ppat.1009797)
Supplement: S1 Text — (DOCX) [file ppat.1009797.s017.docx]

**Supplementary Methods**

**Plasmid preparation**

The *Pfpank2*-pGlux-1 construct has the *Pfpank2*-coding sequence inserted within multiple cloning site (MCS) III of pGlux-1. The plasmid backbone contains the human dihydrofolate reductase (*hdhfr*) gene, which confers resistance to WR99210, as a positive selectable marker. *Pfpank2* is placed under the regulation of the *Plasmodium falciparum* chloroquine resistance transporter (*Pfcrt*) promoter, and upstream of the GFP-coding sequence.

The *Pfpank2* sequence used to generate the *Pfpank2*-pGlux-1 construct was initially amplified from parasite cDNA. Total RNA was purified from saponin-isolated *P. falciparum* parasites (typically 2 $\times$ 10^7^ cells) using the RNeasy Mini Kit (QIAGEN) according to the manufacturer’s protocol for purifying total RNA from animal cells. The optional 15 min DNase I incubation was included to eliminate residual genomic DNA. Complementary DNA (cDNA) was synthesised from this total RNA sample using SuperScript II Reverse Transcriptase (ThermoFisher) with an Oligo(dT)_12-18_ primer and RNaseOUT Recombinant Ribonuclease Inhibitor (ThermoFisher), all according to the manufacturer’s protocol. The *Pfpank2*-specific sequence was then amplified from cDNA using Platinum *Pfx* DNA polymerase (ThermoFisher) with the oligonucleotide primers listed in **S2 Table**. The *Pfpank2*-coding sequence was then inserted into pGlux-1 using the In-Fusion cloning (Clontech) method. Before cloning, the pGlux-1 plasmid was linearised by sequential digestions with *Xho*I (ThermoFisher) and subsequently *Kpn*I (New England Biolabs), according to each manufacturer’s recommendation. The In-Fusion reaction was set up with the In-Fusion Dry-Down PCR Cloning Kit, essentially as described in the manufacturer’s protocol.

The *Tg*PanK1-mAIDHA and *Tg*Pank2-mAIDHA expressing lines were generated using a CRISPR/Cas9 based genome editing approach as previously described in Shen *et al.* [1]. Guide RNA (gRNA) sequences that would enable Cas9 to complex with the RNA to cut close to the 3’ end of either the *Tgpank1* or *Tgpank2* gene were incorporated into pSAG1::Cas9-U6::sgUPRT (Addgene plasmid #54467 [1]) by utilising the Q5 site-directed mutagenesis kit (New England Biolabs). This included performing an initial PCR incorporating the gRNA into the vector and subsequently circularising the vector. This vector encodes both the gRNA and the Cas9-GFP. A gBlock (IDT) containing the mAIDHA construct [2] was also amplified using gene-specific primers with homologous ends to the locus targeted by the gRNA to enable homologous repair following the Cas9-induced double-stranded break at the target locus. The gRNA-expressing pSAG1::CAS9-U6::sgUPRT construct for each *Tgpank* gene was transfected with the corresponding mAIDHA gBlock fragment into RH strain TATiΔ*Ku80*:TIR1 tachyzoite-stage parasites [2] also expressing a tandem dimeric (td) Tomato red fluorescent protein. The GFP expression from the Cas9-GFP enabled the use of flow cytometry to select GFP positive cells to establish a clonal population two days after transfection. Transformants were identified by PCR screening (all primers are listed in **S2 Table** and gBlocks are listed in **S3 Table**).

The *Tg*PanK1-GFP/*Tg*PanK2-mAIDHA expressing line was generated utilising the CRISPR/Cas9 approach as described above. A gBlock (IDT) containing the TEV-GFP construct was amplified using gene-specific primers with homologous ends to the *Tgpank1* locus targeted by the gRNA to enable homologous repair. This was transfected with the gRNA-expressing pSAG1::CAS9-U6::sgUPRT construct for *Tgpank1* into the *Tg*PanK2-mAIDHA line that had been previously created. GFP positive cells were selected for as described above and a clonal line was identified by PCR screening (all primers are listed in **S2 Table** and gBlocks are listed in **S3 Table**). The *Tg*PanK1-HA/*Tg*PanK2-GFP expressing line was generated in RH strain TATiΔ*Ku80* parasites using the same CRISPR/Cas9 approach with the TEV-HA gBlock and TEV-GFP gBlock (IDT).

The complementation lines *Tg*PanK1-mAIDHA^+^*^Sa^*^PanK-Ty1^ and *Tg*PanK2-mAIDHA^+^*^Sa^*^PanK-Ty1^ were created by expressing Ty1-tagged *Staphylococcus aureus* type II pantothenate kinase (*Sapank*) in the *Tg*PanK1-mAIDHA and *Tg*PanK2-mAIDHA *T. gondii* strains. Briefly, the *Sapank* open reading frame was PCR amplified using a gBlock encoding *Sapank* that had been codon-optimised for *T. gondii* expression. The resultant PCR product was digested with *Bgl*II and *Avr*II and ligated into the equivalent sites of the vector pUBTTY. The pUBTTy was modified from the vector pBTTy, described previously [3], which contains a phleomycin resistance marker, and an expression cassette containing the *T. gondii* α-tubulin promoter and a Ty1 epitope tag. The UPRT flank was digested from the vector pUgCTH3 [4] with *Apa*I and *Hin*dIII and ligated into the equivalent sites of pBTTy to generate pUBTTy. The *Sapank*-Ty1 containing pUBTTy vector was linearised and transfected into the lines expressing *Tg*PanK1-mAIDHA and *Tg*PanK2-mAIDHA. Transformants were subsequently selected using phleomycin (50 µg/mL) in DMEM supplemented with 10 mM HEPES and 10 µg/mL gentamicin, pH 7.6, for 4 days, as described previously [5]. Phelomycin-resistant parasites were cloned using fluorescence-activated cell sorting, and subsequently cultured in complete RPMI-1640.

**Preparation of cells for microscopy**

Coverslip-bound *P. falciparum* infected red blood cells were first prepared by washing (500 $\times$ *g*, 5 min) parasite-infected erythrocytes (5 − 10% parasitaemia) once and resuspending them at ~2% haematocrit in 137 mM NaCl, 2.7 mM KCl, 10 mM phosphate buffer, pH 7.4 (phosphate buffered saline; PBS). Next, 1 − 2 mL of the suspension was added to a polyethylenimine (PEI)-coated coverslip placed within a well of a 6-well plate. Plates were incubated (with shaking) for 15 min at room temperature and unbound cells were subsequently washed off the coverslips with PBS (2 mL per well, with a 2 min shaking incubation followed by aspiration). Cells were then fixed with 1 mL of PBS containing 4% (w/v) paraformaldehyde (Electron Microscopy Services) and 0.0075% (w/v) glutaraldehyde (30 min at room temperature). The fixative was then aspirated, and the coverslips washed in PBS three times as described above, before they were rinsed in water and dried. A drop of SLOWFADE (Invitrogen) containing the nuclear stain 4’,6-diamidino-2-phenylindole (DAPI) was added to the centre of the coverslips. Finally, each coverslip was inverted onto a microscope slide, sealed with nail polish and used for confocal imaging.

For *T. gondii* immunofluorescence assays, parasites were inoculated onto HFF-coated coverslips and allowed to proliferate overnight. Parasites were fixed in 3% (w/v) paraformaldehyde in PBS, permeabilised in 0.25% (v/v) Triton X-100 in PBS and blocked in 2% (w/v) bovine serum albumin in PBS. Parasites were incubated in mouse anti-Ty1 primary antibodies (1:200 dilution; [6]) and goat anti-mouse AlexaFluor 488-conjugated secondary antibodies (1:250 dilution; ThermoFisher, catalogue number A11029).

**Denaturing polyacrylamide gel electrophoresis**

Saponin-isolated *P. falciparum* parasites (typically ~10^8^ cells) were centrifuged (15,850 $\times$ *g*, 30 s) and the supernatant was removed. The pellet was resuspended in 200 µL of lysis buffer (1 $\times$ mini cOmplete protease inhibitor cocktail (Roche), 1 $\times$ NuPAGE LDS sample buffer (ThermoFisher), 1 $\times$ NuPAGE sample reducing agent (ThermoFisher), 50 – 60 units of benzonase nuclease (Novagen) and 7.5 – 10 mM of MgCl_2_), mixed well by vortexing and then incubated at 95 °C for 10 min. The sample was then centrifuged (16,000 $\times$ *g*, 30 min) to pellet the haemozoin before the supernatant was used for gel electrophoresis. Samples (10 µL) were subsequently loaded into separate wells of a NuPAGE 4 – 12% or 12% Bis-Tris protein gel (1.0 mm, 12 wells; ThermoFisher) alongside 5 µL of SeeBlue Plus2 pre-stained protein standards (ThermoFisher).

*T. gondii* tachyzoites, either freshly egressed from host HFF cells or mechanically egressed through a 26-gauge needle, were filtered through a 3 µm polycarbonate filter. Tachyzoites (typically 1.5 $\times$ 10^7^ cells), were subsequently centrifuged (12,000 $\times$ *g*, 1 min). The supernatant was aspirated and the pellet was resuspended in 30 µL of 1 $\times$ NuPAGE LDS sample buffer (ThermoFisher). The sample was mixed by vortexing and incubated at 95 °C for 10 min. Samples were then frozen at -20 °C or used immediately for gel electrophoresis. Samples (10 – 20 µL) were subsequently loaded into separate wells of a NuPAGE 4 – 12% Bis-Tris protein gel (1.0 mm, 12 wells; ThermoFisher) alongside 5 µL of Novex Sharp Pre-Stained Protein Standard (Invitrogen). Where relevant, parasites were incubated in 100 µM idole-3-acetic acid (IAA) or in a 0.1% (v/v) ethanol vehicle control for the specified times prior to sample preparation.

Electrophoresis of *P. falciparum* and *T. gondii* samples was performed in 1 $\times$ NuPAGE 2-(*N*-morpholino)ethanesulfonic acid (MES) sodium dodecyl sulfate (SDS) running buffer (ThermoFisher) at 200 V for 30 – 35 min. Separated proteins were transferred (35 V for 1.5 h or 30 V for 1 h depending on the transfer system) to a nitrocellulose membrane (ThermoFisher) in 1 $\times$ NuPAGE transfer buffer containing 10% (v/v) methanol. The membrane was then blocked in a solution containing 4% (w/v) skim milk powder in Tris buffered saline (TBS) or PBS with shaking, either overnight at 4 °C or for 1 – 2 h at room temperature.

The primary antibodies used in this study included mouse anti-GFP monoclonal antibody (0.4 µg/mL final concentration; Roche, Sigma catalogue 11814460001), rat anti-HA monoclonal antibody (1.6 µg/mL final concentration; Sigma, clone 3F10, catalogue A11867431001), mouse anti-Ty1 monoclonal antibody (1:1000 dilution; [6]), and a pan-specific anti-14-3-3 rabbit polyclonal antibody (0.25 µg/mL final concentration; Abcam). The secondary antibodies used for the *P. falciparum* blots were a goat anti-mouse horseradish peroxidase (HRP)-conjugated antibody and a goat anti-rabbit HRP-conjugated antibody (both 0.08 µg/mL final concentration; Santa Cruz Biotechnology). The secondary antibodies used for *T. gondii* experiments were goat anti-mouse HRP-conjugated antibody (0.4 – 0.8 µg/mL final concentration; Abcam), goat anti-rabbit HRP-conjugated antibody (0.1 µg/mL final concentration; Abcam), and goat anti-rat HRP-conjugated antibody (0.1 µg/mL final concentration; Abcam).

All antibodies were diluted in 4% (w/v) skim milk in TBS or PBS. After each antibody incubation, membranes were washed at least three times (5 – 10 min each) in fresh 0.05 or 0.1% (v/v) Tween 20 in TBS or PBS.

**Native polyacrylamide gel electrophoresis**

In order to detect the presence and abundance of protein(s) of interest in their native conformation, parasite samples were subjected to blue native gel electrophoresis. Briefly, saponin-isolated trophozoite-stage *P. falciparum* parasites (4 – 8 $\times$ 10^8^ cells) were centrifuged (15,850 $\times$ *g*, 30 s) and the supernatant removed from the pellet. The parasites were then resuspended by vortexing in 200 µL of lysis buffer containing 1 $\times$ mini cOmplete protease inhibitor cocktail (Roche), 1 $\times$ NativePAGE sample buffer (ThermoFisher), 0.5% (w/v) digitonin, 2 mM EDTA, 50 – 60 units of benzonase nuclease (Novagen) and 7.5 – 10 mM of MgCl_2_, and incubated with tumbling end-over-end at 4 °C. The lysis preparation was then centrifuged at 16,000 $\times$ *g* for 30 min at 4 °C and the supernatant immediately used for gel electrophoresis. Prior to electrophoresis, NativePAGE 5% G-250 sample additive (ThermoFisher) was added to each sample supernatant to a final concentration of 0.125% (w/v) and mixed by vortexing. Samples (typically 10 µL) were then loaded into the wells of a NativePAGE 4 – 16% Bis-Tris protein gel (1.0 mm, 10 wells; ThermoFisher). NativeMark unstained protein standards (5 µL, ThermoFisher) were loaded into the gel alongside the samples to allow for protein mass determination. Electrophoresis was carried out at 4 °C according to the manufacturer’s protocol for detergent-containing samples to be used for western blotting. At the end of the run, the proteins within the gel were transferred as described for denaturing western blot above to a methanol-primed 0.45 µm PVDF membrane (GE healthcare). At the end of the transfer, proteins were fixed to the membrane by a 15 min incubation in 10% (v/v) acetic acid and briefly rinsed in water. In order to visualise the ladder, the membrane was very briefly (~5 s) de-stained in absolute methanol and rinsed in water before it was blocked overnight in 4% (w/v) skim milk powder in PBS at 4 °C with shaking.

*T. gondii* tachyzoites freshly or mechanically egressed from their host HFF cells were filtered through a 3 µm polycarbonate filter. Tachyzoites (typically 1.5 $\times$ 10^7^ cells) were subsequently centrifuged (12,000 $\times$ *g*, 1 min). The supernatant was removed and the pellet was resuspended by vortexing in 30 µL of lysis buffer containing 1 $\times$ mini cOmplete protease inhibitor cocktail (Roche), 1 $\times$ NativePAGE sample buffer (ThermoFisher), 10% (v/v) Triton X-100, 2 mM EDTA, and incubated with intermittent perturbation on ice for 30 min. The lysate was then centrifuged at 20,000 $\times$ *g* for 30 min at 4 °C and the supernatant was either stored at -20 °C or used immediately for gel electrophoresis. Sample preparation, electrophoresis and transfer was carried out as described for *P. falciparum.* Where anti-HA blotting was required on a PVDF membrane, after electrophoresis of the samples the membrane was placed in TBS containing 0.05% (v/v) Tween 20 overnight, then blocked in 4% (w/v) skim milk powder in TBS at room temperature for a minimum of 1 h the next day before probing with the anti HA antibody.

**Immunoprecipitation**

Briefly, saponin-isolated *P. falciparum* trophozoites were resuspended in 500 µL of lysis buffer containing 1 $\times$ mini cOmplete protease inhibitor cocktail (Roche) or 1 $\times$ Halt protease inhibitor cocktail (EDTA-free; ThermoFisher), GFP-Trap wash buffer (10 mM Tris/Cl, pH 7.5, 150 mM NaCl and 0.5 mM EDTA), 0.5% (w/v) digitonin, 50 – 60 units of benzonase nuclease (Novagen) and 3 – 4 mM of MgCl_2_. The pellet was resuspended well by vortexing and the suspension was incubated (30 – 60 min) with tumbling end-over-end at 4 °C. Subsequently, the suspension was centrifuged (16,000 $\times$ *g*, 30 min, 4 °C) and the supernatant used for GFP-Trap binding. Prior to immunoprecipitation, 25 µL (for each lysate) of GFP-Trap-agarose bead slurry was primed by three washes (2,500 $\times$ *g*, 2 min, 4 °C) in 500 µL of GFP-Trap wash buffer. The supernatant was removed from the beads at the end of the third wash and 450 – 500 µL of the lysate generated in the parasite lysis step was applied to the beads. In some experiments, 50 µL of the total lysate was collected for western blotting. This suspension was then incubated for 1 h at 4 °C with tumbling end-over-end. At the end of the incubation, the bead suspension was centrifuged (2,500 $\times$ *g*, 2 min, 4 °C) and the supernatant was discarded. In some experiments, 50 µL of this supernatant was collected for use in western blots as the unbound fraction. The proteins bound to the beads were washed 3 $\times$ (2,500 $\times$ *g*, 2 min, 4 °C) in GFP-Trap wash buffer with or without 1 $\times$ mini cOmplete protease inhibitor cocktail or 1 $\times$ Halt protease inhibitor cocktail (EDTA-free). After removing the supernatant at the end of the third wash, the beads were resuspended in GFP-Trap wash buffer (typically 200 – 300 µL) and aliquots of these were used for downstream experiments.

*T. gondii* GFP-tagged proteins were purified using the GFP-Trap approach mentioned above, and the HA-tagged proteins were purified using anti-HA Affinity Matrix (Roche) following the manufacturer’s instructions with some modifications. Egressed *T. gondii* tachyzoites were filtered through a 3 µm polycarbonate filter. Parasites (~10^7^-10^8^ for each line) were centrifuged (1,500 $\times$ *g*, 10 min, 4 °C). The supernatant was aspirated, and the cells were resuspended in 1 mL of PBS and centrifuged (12,000 $\times$ *g*, 1 min, 4 °C). After aspiration, lysis buffer (1 mL) containing 1 $\times$ mini cOmplete protease inhibitor cocktail (Roche), wash buffer (10 mM Tris/Cl, pH 7.5, 150 mM NaCl and 0.5 mM EDTA), and 1% (v/v) Triton X-100 was added to the remaining cells. The pellet was resuspended and the suspension was incubated (1 h) with tumbling end-over-end at 4 °C. Subsequently, the suspension was centrifuged (21,000 $\times$ *g*, 30 min, 4 °C). Prior to immunoprecipitation, 25 µL of GFP-Trap-agarose bead slurry, or 25 µL of anti-HA Affinity Matrix were washed three times in 500 µL of wash buffer (with 1% (v/v) Triton X-100 in the anti-HA wash buffer), with centrifugation at 2,500 $\times$ *g*, 2 min, 4 °C between washes. The supernatant was removed from each aliquot of the beads after the third wash, and 450 µL of the parasite lysate was applied to the washed beads. A 50 µL aliquot of the total lysate was collected for western blotting as the ‘total’ fraction. The lysate/bead suspension was incubated for at least 1 h at 4 °C with tumbling end-over-end. At the end of the incubation, the bead suspension was centrifuged (2,500 $\times$ *g*, 2 min, 4 °C), and 50 µL of this supernatant was collected for use in western blots as the ‘unbound’ fraction. The proteins bound to the beads were washed 3 $\times$ in wash buffer with centrifugation at 2,500 $\times$ *g*, 2 min, 4 °C between washes. After removing the supernatant at the end of the third wash, the beads were resuspended in wash buffer (typically 100 – 300 µL) and aliquots of these were used for downstream experiments. Alternatively, 100 µL of 1 $\times$ NuPAGE LDS sample buffer (ThermoFisher) was added to the samples to elute proteins from the beads and generate the ‘bound’ fractions.

**Mass spectrometry of immunoprecipitated samples**

The immunoprecipitated proteins were processed and identified through mass spectrometry (MS) analysis at the Australian Proteomics Analysis Facility (APAF). First, the loading buffer in the samples were separated from the proteins through a short one-dimension gel electrophoresis. The samples were denatured at 95 °C for 10 min and 2 $\times$ 15 µL of each sample was loaded into the lanes of a 12% iGel protein gel (1.0 mm, 12-well; NuSep). The gel was run at 15 mA for 25 min and subsequently washed with a solution containing 10% (v/v) methanol and 7% (v/v) acetic acid for 15 min. The gel was then washed with a fixant for 90 min and stained overnight in Coomassie. The band corresponding to the proteins was subsequently excised and de-stained with ammonium bicarbonate/acetonitrile (ACN). The protein samples were then reduced with 25 mM dithiothreitol (DTT) at 60 °C for 30 min and alkylated with 55 mM iodoacetamide before an in-gel protein digestion was performed overnight using 200 ng trypsin. The peptides generated were extracted from the gel with bath sonication and ACN/formic acid (FA), dried and then reconstituted in 30 µL of loading buffer.

The peptide samples were then subjected to 1D nano liquid chromatography tandem mass spectrometry (Nano-LC-ESI MS/MS) analysis. Sample (10 µL) was injected onto a peptide trap (Halo C18, 150 µm $\times$ 5 cm) for pre-concentration and desalted with 0.1% (v/v) FA, 2% (v/v) ACN at 4 µL/min for 10 min. The peptide trap was then switched into line with the analytical column. Peptides were subsequently eluted from the column using a linear solvent gradient, with steps, from H_2_O:ACN (98:2; $+$ 0.1%, v/v, FA) to H_2_O:ACN (2:98; $+$ 0.1%, v/v, FA) with constant flow (600 nL/min) over an 80 min period. The liquid chromatography eluent was subjected to positive ion nanoflow electrospray MS analysis in an information-dependent acquisition mode (IDA). In the IDA mode, a time-of-flight MS survey scan was acquired (*m/z* 350-1500, 0.25 s), with the twenty largest multiply charged ions (counts $>$150) in the survey scan sequentially subjected to MS/MS analysis. MS/MS spectra were accumulated for 100 ms (*m/z* 100 – 1500) with rolling collision energy.

The peptides from the MS analysis were identified by comparing their amino acid sequences against an annotated protein database for *P. falciparum* 3D7 strain (version 28; PlasmoDB) using the ProteinPilot Software (version 4.2; SCIEX) at a detection threshold of $>$1.30 (95.0% confidence).

**Fluorescent *T. gondii* proliferation assay**

Fluorescent *T. gondii* proliferation assays were performed as previously described [4,7] with RH Δ*Ku80*:TIR1 strain *T. gondii* parasites. Black optical bottom 96-well plates containing confluent HFF host cells were washed with PBS ($\times$ 2). Complete RPMI-1640 (100 µL) with or without 200 µM of IAA supplementation (2 $\times$ final concentration), or with 10 µM pyrimethamine (2 $\times$ final concentration) for the ‘no growth’ control, were added to the relevant wells. Fluorescent parasites (parental line, *Tg*PanK1-mAIDHA, *Tg*PanK2-mAIDHA, *Tg*PanK1-mAIDHA^+^*^Sa^*^PanK-Ty1^ or *Tg*PanK2-mAIDHA^+^*^Sa^*^PanK-Ty1^) were plated in each well (100 µL, 2000 parasites) in triplicate. Plates were incubated at 37 °C in a 5% CO_2_ humidified incubator. Fluorescent measurements (Excitation filter, 540 nm; Emission filter, 590 nm) were taken up to two times a day over 7 days with the FLUOstar OPTIMA Microplate Reader (BMG LABTECH), and the proliferation of the fluorescent parasites was measured over this time. Values for the no growth control were considered as background values and were subtracted from the experimental values during data processing.

**REFERENCES**

1. Shen B, Brown KM, Lee TD, Sibley LD. Efficient gene disruption in diverse strains of *Toxoplasma gondii* using CRISPR/CAS9. MBio. 2014;5: e01114–14. doi:10.1128/mBio.01114-14

2. Brown KM, Long S, Sibley LD. Plasma membrane association by N-acylation governs PKG function in *Toxoplasma gondii*. MBio. 2017;8. doi:10.1128/mBio.00375-17

3. Brooks CF, Johnsen H, van Dooren GG, Muthalagi M, Lin SS, Bohne W, et al. The *Toxoplasma* apicoplast phosphate translocator links cytosolic and apicoplast metabolism and is essential for parasite survival. Cell Host Microbe. 2010;7: 62–73. doi:10.1016/j.chom.2009.12.002

4. Rajendran E, Hapuarachchi SV, Miller CM, Fairweather SJ, Cai Y, Smith NC, et al. Cationic amino acid transporters play key roles in the survival and transmission of apicomplexan parasites. Nat Commun. 2017;8: 14455. doi:10.1038/ncomms14455

5. Messina M, Niesman I, Mercier C, Sibley LD. Stable DNA transformation of *Toxoplasma gondii* using phleomycin selection. Gene. 1995;165: 213–217. doi:10.1016/0378-1119(95)00548-k

6. Bastin P, Bagherzadeh A, Matthews KR, Gull K. A novel epitope tag system to study protein targeting and organelle biogenesis in *Trypanosoma brucei*. Mol Biochem Parasitol. 1996;77: 235–239. doi:10.1016/0166-6851(96)02598-4

7. Gubbels MJ, Li C, Striepen B. High-throughput growth assay for *Toxoplasma gondii* using yellow fluorescent protein. Antimicrob Agents Chemother. 2003;47: 309–316. doi:10.1128/AAC.47.1.309-316.2003
