# Supplementary material for: A novel heteromeric pantothenate kinase complex in apicomplexan parasites
Source: PLoS Pathog. 2021 Jul 29;17(7):e1009797. doi: 10.1371/journal.ppat.1009797 (PMC8366970; doi:10.1371/journal.ppat.1009797)
Supplement: Attachment — Submitted filename: Response to Reviewers.pdf [file ppat.1009797.s018.pdf]

Response to the editors' and reviewers' comments

We have followed the advice of the editors and paid specific attention to the points they highlighted. We have carried out the two key experiments that were requested. We have also included a point-by-point response to the comments from all four reviewers. We have now also included an Author Summary (lines 32-48). For clarity, small changes to the manuscript, such as renumbering of the supplementary figures, reformatting of references and minor corrections/improvements are not highlighted in the "manuscript with changes highlighted".

As you will see from the comments, all of the reviewers appreciate the work, the quality of the data and the contribution to the field. Reviewers 1, 2 and 3 suggest making relatively minor revisions, most of which are aimed at enhancing and clarifying different aspects of the results. Reviewer 4 made several suggestions and brought to your attention an article that was published today concerning PfPanK1 activity. In your response to the suggestions made by Reviewer 4, please pay specific attention to these points:

- Provide specific activity units in Figure 1c (as done in Fig 2).

Response: We have not changed the units of Figure 1c. We have provided an explanation for this decision next to the assessor's specific comment.

- Determine the PanK activity of material (IP or lysate) from the Tg line TgPanK1-GFP/TgPanK2-mAIDHA after inducing knockdown of PanK2.

Response: This is a very good suggestion. We have carried out this experiment and the new data is presented in Figure 5 (new figure). We demonstrate that knockdown of *Tg*PanK2 results in the rapid degradation of *Tg*PanK2, but has no effect on the abundance of *Tg*PanK1-GFP. Immunoprecipitated *Tg*PanK1-GFP possesses PanK activity when *Tg*PanK2 is present, but is devoid of activity when *Tg*PanK2 is knocked down. This indicates that *Tg*PanK2 is required for *Tg*PanK activity in the parasite, and supports our overall hypothesis that apicomplexan PanK is a heteromeric complex, requiring both PanK1 and PanK2 proteins in order to function. New experiment and figure legend can be found on lines 353-364 and 371-387.

- Provide information about whether Tg14-3-3I can be detected with pan anti-14-3-3 in the TgPanK complex. Reviewer 1 also brought up this point.

Response: We have now carried out this experiment and show that although the pan anti-14-3-3 antibody detected a protein consistent with the size of *Tg*14-3-3 in *T. gondii* lysates, the protein was not detected in the purified *Tg*PanK complex. These new data have been included in Figure S10 and discussed in lines 255-259 and 453-458.

Reviewer's Responses to Questions

Part I - Summary

I have some minor considerations which I list out below:

1. In Figure 2C, right panel, in which we observe the immunoprecipitation of the Pf14-3-3 protein. Here I think it is important to include the Western blots that show that

immunoprecipitation of the GFP tagged PfPanK1 and PfPank2 for the experiment as well as the GFP control. Please provide all relevant panels of the immunoprecipitation experiment so that the evaluation of the experiment is complete.
Response: The additional information about Figure 2c (right panel) is provided in Figure S2. It is not clear if the reviewer is requesting that we move Figure S2 to Figure 2 or if the reviewer overlooked Figure S2 in the supplementary section. Our preference is to leave it in the supplementary section, but we are happy to move it to the main text if requested by the editor. We have now included explicit reference to the data in Figure S2 in the legend of Figure 2c.

2. The authors speculate that the TgPanK1 and TgPanK2 complex also harbors another yet unidentified protein due to the increase in molecular weight. Did the authors try the pan 14-3-3 antibody on these T. gondii extracts? If a corresponding 14-3-3 homolog does not exist in T. gondii, it would be good to mention this in the text. If one does exist, it would be good to discuss whether the 14-3-3 antibody detected anything within the TgPanK1/2 complex, even if it was a negative result.
Response: We have now carried out this experiment. *Tg*14-3-3 appears to be absent from the *Tg*PanK complex. This information has been included in the manuscript (Figure S10, lines 255-259 and 453-458).

3. PanOH-A, PanOH-B and CJ-A harbour mutations in PfPanK1 and has been previously characterized. Nevertheless, it would have been helpful in the text to list the mutations in PfPanK1 that are present within these strains.
Response: These specific *Pf*PanK1 mutations have now been included in the text (lines 160-163).

4. It is clear that TgPanK1 and TgPanK2 are essential for proliferation. Would the authors be able to provide any additional information about the nature of the death using cell biology techniques?
Response: We are very interested in looking into this, but we feel that answering this question is beyond the scope of the present study. Because CoA is involved in so many biochemical reactions (estimates have indicated that approximately 9% of all biochemical reactions involve CoA or CoA thioesters; PMID 18173393), parasite inhibition due to CoA starvation is likely to be multifactorial. An additional complication in establishing the cause of proliferation inhibition is that although *Tg*PanK levels decrease very rapidly upon induction of knockdown, we do not yet have temporal data on CoA levels following *Tg*PanK knockdown. The parasite strains we have generated in this study will allow us to start investigating this question in the future.

5. Can the authors comment on why they used SaPanK for the compensate for the PanK loss in the conditional knockdown vs using PfPanK1 or PfPanK2?
Response: We elected not to use the *P. falciparum* counterparts for a number of reasons: (i) since the interspecies protomers may not form a heteromeric complex, we would have had to express both *Pf*PanK1 and *Pf*PanK2 simultaneously; (ii) the *Tg*14-3-3 may not have been able to associate with the *Pf*PanK complex when expressed in *T. gondii;* and (iii) expressing an orthologue with a known function allows us to conclude that the complemented functionality is due to PanK activity rather than a hitherto unknown function of the complex.

We therefore chose to express the *Sa*PanK because it harbours a well-characterised, homodimeric (i.e you only need to express the one gene), type-II PanK. As a type-II PanK, it more closely resembles eukaryotic PanKs than it does prokaryotic PanKs. Importantly, *Sa*PanK is also refractory to negative feedback inhibition by CoA. High concentrations of CoA within *T. gondii* would therefore not prevent the enzyme from functioning. We have included this rationale in the manuscript (lines 310-316).

Reviewer #2: The authors present a comprehensive analysis of a previously unreported heterodimeric PanK present in two apicomplexan parasites. A combination of biochemical, proteomic, and cell biological techniques deomstrates the activity of this new form of PanK complex and confirms that both PanK proteins are essential for PanK activity and parasite survival. Overall, the work is thorough and complete. It is well presented, and the data supports the authors' conclusions.

Given the quality of the work it is a bit disappointing that the explanation of the broader significance of the findings is not more explicit. It is implied that the study is of wider importance because the novel, heterodimeric nature of the apicomplexan PanKs might provide insight into the purpose of "non-functional" and non-canonical PanKs found in other organisms. This idea is not developed further in the discussion with, for instance, a comparison of the structure of "non functional" PanKs in apicomplexans and other organisms and a discussion of whether heteromeric PanKs might be biologically important beyond the apicomplexans. The importance of the heteromeric PanKs in anti-parasitic drug development is only briefly mentioned, with no discussion on the impact this new PanK structure might have in the context of known inhibitors and their development as new antimalarials. A revision of the Introduction and Discussion to provide a deeper insight into these topics would provide the non-expert reader a better understanding of why this previously unreported form of PanK is of general significance.
Response: We have not elaborated further on the non-functional PanKs and the potential significance of the complex to drug development. We had deliberately kept the discussion on these topics brief because we believe that a detailed discussion would be too speculative. This would be particularly true if we attempted to discuss "the structure of non-functional PanKs in apicomplexans" since there is currently no structure available for these proteins. Such a discussion, therefore, is not really possible.

Reviewer #3: The manuscript "A novel heteromeric pantothenate kinase complex in apicomplexan parasites" by Tjhin et al describes functional characterization of a unique heteromeric pantothenate kinase in P. falciparum and T. gondii which is formed by association of PanK1 and PanK2 proteins. By using a combination of fluorescent microscopy, immunoprecipitation, mass spectroscopy and SDS-PAGE or native PAGE the authors demonstrate that both PfPanK1 and PfPanK2, along with another protein, Pf14-3-3I are part of pantothenate kinase complex. This was further supported by studies involving episomal expression of PfPanK2 in pantothenate kinase mutant strains of P. falciparum. Similar observations regarding heteromeric complex of PanK1 and PanK2 were made in another parasite T. gondii by using an auxin inducible degron fusion and immunoprecipitation techniques. A combination of knockdown and heterologous complementation by S. aureus type II Pank established the essentiality of PanK1 and PanK2 for the proliferation of T. gondii parasites.

P. falciparum and T. gondii are important pathogens causing human disease and there is an urgent need to discover drugs with novel mechanism of action for controlling these diseases. Pantothenate kinase has been suggested to be a drug target in a number of bacterial pathogens. The results of this study suggest that pantothenate kinase is a potential drug target in these two parasites as well. The formation of heteromeric enzyme complex by association of PanK1 and PanK2 also explains why individually expressed PanKs in heterologous systems had not shown any catalytic activity in a number of earlier studies. This is a scientifically sound study which has clearly established the essential nature and catalytic activity of pantothenate kinase in P. falciparum and T. gondii. The conclusions drawn have been duly supported with proper experimentation. All the experiments have been designed logically with proper controls whenever required. The publication of this study will open up new avenues in pantothenate kinase research and will help in studies involving functional characterization of hitherto uncharacterized pantothenate kinases in prokaryotes and eukaryotes.

Reviewer #4: The authors aimed at demonstration of the significance of the presence of two PanK in apicomplexan parasites. They demonstrated that PfPanK1 and 2 likely form a dimer of 240 kDa by BN-PAGE, and that the complex possesses kinase activity toward pantothenate. The authors reciprocally identified PfPanK1 and PfPanK2 by immunoprecipitation from GFP-PfPanK1 or GFP-PanK2-expressing transformant lines with comparable efficiency. Notably 14-3-3I (and also M17 leucyl aminopeptidase) were also detected at the significant level, although its significance was not further examined. They created GFP-PfPanK expressing lines also in PanK-inhibitor-resistant lines (PanOH-A, PanOH-B, and CJ-A), which carry mutations in PfPanK1, but not PfPanK2 (?), gene. The ranking of the strains based on the PanK activity detected in the lysate of both control and GFP-PanK1 expressing tranformant line in wildtype and three resistant strains are the same as the ranking when immunoprecipitated samples were used as enzyme source, which only circumstantially supported PanK1 and GFP-PanK2 interaction. However, this observation can be also explained by possible up- or down-regulation of PfPanK1/2 gene expression, post-translational modifications, and interaction with other accessary proteins (such as those detected by MS analysis of the immunoprecipitated samples).
Response: We disagree that the evidence from this experiment is circumstantial. We believe that this experiment provides direct evidence. When the *Pf*PanK complex is purified by immunoprecipitating *Pf*PanK2-GFP (with GFP-Trap) the effect of *Pf*PanK1 mutations on PanK activity are indistinguishable from those observed in whole cell lysates. In our view, this provides robust evidence that immunoprecipitation of *Pf*PanK2-GFP simultaneously pulls down *Pf*PanK1 in the complex and that the activity observed in the immunoprecipitated samples are the same as those observed *in situ*. Additionally, we demonstrate that *Tg*PanK1 and *Tg*PanK2 associate in *T. gondii*, and that both contribute to parasite proliferation and PanK activity. Together, our data provide considerable evidence for the “significance of the presence of two PanKs in apicomplexan parasites”.

Part II – Major Issues: Key Experiments Required for Acceptance
Please use this section to detail the key new experiments or modifications of existing experiments that should be absolutely required to validate study conclusions.

Generally, there should be no more than 3 such required experiments or major modifications for a "Major Revision" recommendation. If more than 3 experiments are necessary to validate the study conclusions, then you are encouraged to recommend "Reject".

Reviewer #1: None.
Reviewer #2: (No Response)
Reviewer #3: (No Response)
Reviewer #4: This is carefully designed and well executed experiments in order to understand the conundrum that some organisms possess two PanK-like proteins, using two representative apicomplexan parasites, Pf and Tg, as examples. Discovery of the complex formation and essentiality of the both PanK isotypes for PanK activity and proliferation (though the essentiality in Pf was previously demosntrated) is a major contribution of the study to the fields. However, how the regulation of PanK activity by PanK2, e.g., via modulation of stability or protein complex conformation, remains totally undetermined. Furthermore, its relevance to pathogenesis of the two parasites are also not properly discussed. These two issues are more or less the critical points that need to be clarified.
Response: We have not claimed that PanK2 acts to regulate PanK activity. The key finding in our study is that PanK1 and PanK2 form a functional unit for PanK activity. We also put forward the hypothesis that the two protomers come together to create a single active site (instead of the two active sites formed by homodimer PanKs). We therefore do not believe that the role of PanK2 is a regulatory one, but, rather, it is essential for the formation of a functional complex. We also made no claim that the PanK complex in these two parasites has any relevance to pathogenesis. It is unclear to us why the reviewer believes we should discuss this.

In addition, two additional critical missing points here are: they did not demonstrate stoichiometry of the two isotypes in the complex (e.g., the ratio of the components).
Response: We disagree that information about the stoichiometry of the complex is “critical” for the manuscript. We believe that carrying out experiments to determine the stoichiometry of the isotypes in the complex (which is likely to be 1:1 as they function as dimers) is beyond the scope of the study.

Activity was simply shown as arbitrary (?) radio isotope counts, but not in specific activity (e.g., Fig. 1C), thus it was not evaluated that the complex is as active as detected in the whole cell lysate.
Response: We provide a direct comparison of specific PanK activity in cell lysates *versus* the purified complex in Figure 2b(ii). Comparison of the activity (in µmols pantothenate phosphorylated/10^{12} cells) in whole cell lysates from the Parent line (Figure 2b(i)) with that of the immunoprecipitated complex generated from the same line transfected with *Pf*PanK2-GFP (shown in the same units, Figure 2b(ii)) reveals that the two are indistinguishable (0.211 µmol/10^{12} cells/min for lysate versus 0.177 µmol/10^{12} cells/min for immunoprecipitated complex). We have included a comment about this important point in the manuscript (lines 178-183). To generate the data in Figure 2b(ii), the samples were standardised by counting the number of GFP positive parasites (using FACS, Figure S6a). In addition, to confirm that equivalent amounts of protein were used between experiments and parasite lines, the samples were also subjected to western blots (Figure S6b). Since a

similar standardisation protocol was not necessary for the experiment presented in Figure 1c, it was not carried out. We are therefore unable to convert the units of the data presented in Figure 1c into specific activity units. However, since the comparison the reviewer was interested in is readily accessible from the data in Figure 2b, we feel that changing the units of Figure 1c is not essential.

In addition, PanK activity was not demonstrated using individual isoform of PfPanK1 and 2. The following recent paper demonstrated PanK1 activity using a single E. coli recombinant protein (not as a heterocomplex with PanK2) (Nurkanto et al., Front Cell Inf Microbiol, 2021; doi: 10.3389/fcimb.2021.639065). Thus, the biological significance of the heterodimer of PfPanK can be demonstrated using the two PfPanK recombinants.

Response: We have made no attempt in our study to investigate the individual isoforms of *Pf*PanK1 and *Pf*PanK2 since the immunoprecipitated samples always appeared to contain the two isoforms in complex with each other. We had pointed out in the Discussion that previous attempts (by us and others, e.g. PMID 31534021) to measure PanK activity from heterologously-expressed *Pf*PanK1 have been unsuccessful. The observation by Nurkanto *et al.* (2021) that heterologously-expressed, *in vitro*-solubilised, *Pf*PanK1 is active is surprising and, at face value, appears to contradict the findings in our study. It should be noted, however, that the *Pf*PanK1 kinetic parameters reported by Nurkanto *et al.* (pantothenate K_M = 44.6 μM) are very different to those previously reported (PMID 11278793, PMID 24333332 and PMID 29614109) for the kinetics of *Pf*PanK from parasite lysates (K_M ~ 300 nM), suggesting that the activity reported by Nurkanto *et al.* for *Pf*PanK1 may not reflect the endogenous *Pf*PanK activity. We have now commented on this in the Discussion (lines 396-409). It is also worth noting that we demonstrate that when *Tg*PanK1 is immunoprecipitated following *Tg*PanK2 knockdown (Figure 5), no PanK activity can be detected, indicating that *Tg*PanK2 is required for *Tg*PanK1-dependent PanK activity.

Specific points:

Fig. 1b: Both PfPanK1-GFP and PfPanK2-GFP appear to be present as multiple truncated and untruncated forms (e.g., PfPanK1-GFP, ca 30, 38, 49, and 60kDa and 70-80 kDa untruncated form)(PfPanK2-GFP, ca 40, 60 kDa and -100kDa). It is important to know if such truncated forms also retains binding ability with PanK1 and 14-3-3I, and other possible accessory factors, and moreover if the truncated forms are also retain activity. The authors must address those points using recombinant proteins.

Response: We believe that the truncated forms the reviewer refers to are a by-product of sample processing. We believe that exploring these truncated forms using recombinant proteins (as suggested by the reviewer) constitutes an entirely new study that is unlikely to yield meaningful data. Since these truncated forms have been detected in anti-GFP western blots, it is reasonable to assume that they all retain the GFP tag. Therefore, subtracting the size of the GFP protein from the truncated forms leaves *Pf*PanK1 and *Pf*PanK2 fragments that are approximately 2, 10, 12, 21 and 31 kDa – fragments that are unlikely to possess PanK activity. Importantly, such a study (irrespective of the outcome) would not alter any of the conclusions currently presented in our manuscript. We have therefore not carried out these experiments.

It is also important to note there is an additional complex (?) at -140kDa detected in the sample immunoprecipitated from PfPanK1-GFP only. After all, it is not clear at all which of

the two isotypes is responsible for catalytic and regulatory? activities. Based on Tg KD experiments, it was shown that both isotypes are necessary for Tg proliferation. How about PanK activity? Specific KD of TgPanK1 or 2 reduces PanK activity in a time-dependent fashion?

Response: The experiment suggested by the reviewer (i.e. whether specific knockdown of *Tg*PanK1 or *Tg*PanK2 reduces PanK activity) has been carried out and the results included in the manuscript (Figure 5). When *Tg*PanK2 is knocked down, *Tg*PanK1 remains intact, but PanK activity is not detected. Alongside the PanK activity assays when *Pf*PanK2 is immunoprecipitated from mutant *Pf*PanK1-expressing parasites (Fig. 2b(ii)) and the *Sa*PanK complementation data in the *Tg*PanK1 and *Tg*PanK2 regulatable lines (Fig. 4c), this provides compelling evidence that both PanK1 and PanK2 are required for PanK activity in these parasites.

Significance of interaction with 14-3-3I was intriguing; however, no further evidence for a potential involvement of 14-3-3I in influence on PanK activity, its synthesis and turnover, have not been provided. Neither was for other PanK1/2 associated proteins. Proteome data were not fully utilized and downstream research is rather superficial and sufficient for publication in PLoS Pathogens. They proposed in discussion section that Pf14-3-3I plays a regulatory role in the PfPanK complex. At least, conditional knockdown by GlnS-ribozyme-based conditional KO or destabilizing domain-based KD of Pf14-3-3I can be tried to see if PfPanK1/2 gene expression, protein localization, and function are affected.

Response: Exploring the role of *Pf*14-3-3I in the *Pf*PanK complex is of considerable interest to us. However, given that *Pf*14-3-3I knockdown is likely to have multiple effects in the parasite (due to the protein's ability to interact with various proteins), interpretation of the *Pf*14-3-3I conditional knockout results will likely be very complex. We believe, therefore, that these experiments are beyond the scope of our current manuscript. To avoid speculation, we have removed reference to *Pf*14-3-3I playing a regulatory role in the *Pf*PanK complex (deleted sentence used to be between the sentences on line 449).

Furthermore, since 14-3-3I was not identified by immunoprecipitated from Tg, generalization of the possible interaction of PanK with 14-3-3I in apicomplexa should not be made.

Response: This is a fair point, especially given that new experiments we have conducted to determine whether 14-3-3 is in the *Tg*PanK complex failed to reveal its presence. The discussion pertaining to 14-3-3 has therefore been modified to reflect this point (lines 455-458).

It was argued that the proteome of TgPanK was unsuccessful, but the whole content of the manuscript was intended to generalize the heteromeric formation of PanK for its activity, so it is indeed a pity if the proteomic analysis of TgPanK complex is not included in this study. This referee feels it is needed to include this for the completion of the present study if they generalize the observation for apicomplexa.

Response: The key message from our study is that the two PanK isoforms from both *P. falciparum* and *T. gondii* function as heterodimers in complexes that include other proteins. Whilst knowing the entire composition of the complex is of great interest to us (and is something we will pursue), we do not believe that it is essential for delivering the message of this manuscript.

Part III – Minor Issues: Editorial and Data Presentation Modifications
Please use this section for editorial suggestions as well as relatively minor modifications of existing data that would enhance clarity.

Reviewer #1: Please see Part 1 summary
Response: The comments raised by this reviewer in Part 1 summary have been addressed.

Reviewer #2: I addition to a revision of the Introduction and Discussion mentioned above, I would suggest some editing of the section describing the PfPanK immunoprecipitations. The experiments are excellent but the naming of the recombinant proteins and the multiple uses of GFP tags to pull down different complexes can make the details hard to follow. A minor reworking of this section and figures to emphasize which PanK is being expressed in each cell line (the type of tag is not as important), which protein is being immunoprecipitated, and which is being co-immunoprecipitated, would make the experiments easier to follow and the results easier to interpret. The inclusion of a diagram(s) in Figure 1/2 outlining the experimental approach would be helpful. While the description of the T. gondii immunoprecipitations was clearer, a diagram of these experiments would also be helpful.
Response: We have modified the text to simplify how we refer to the *P .falciparum* parasite lines (e.g. instead of referring to "*Pf*PanK1-GFP-expressing parasites" we simply say "*Pf*PanK1-GFP line". Note that there were many instances of this change throughout the manuscript and we have therefore not highlighted them in the manuscript with changes highlighted. We have also generated a flow diagram (now included in the Supplementary File as Figure S1) which explains, in a simple way, how the samples from both *P. falciparum* and *T. gondii* were generated/processed, what types of experiments the various samples were used for and what figures the results can be found in. We believe that the modifications to the text, as well as the flow diagram, will make the text easier to read and the experiments easier to understand. The first reference to this new figure in the manuscript is highlighted (line 106).

Minor edits:
Line 41-43 should read "Interestingly, many eukaryotes (such as Arabidopsis thaliana 11,12, Mus musculus 13-16 and Homo sapiens 17-21) express multiple PanKs."
Response: Fixed (now on lines 60-62).

Line 61: the comma after PfPank2 isn't needed.
Response: Fixed (now on line 81).

Line 341: "likely" can be deleted.
Response: Fixed (now on line 423).

Figure 2a: the inclusion of M17 leucyl-aminopeptidase as the fourth most abundant protein in the text and in column two is a bit misleading. There are other proteins with more peptides recovered in the PanK2 immunoprecipitation and this should be made clearer.

Response: : We now clarify in the legend to Figure 2a that "Proteins detected in the untagged GFP line or wild-type 3D7 parasite immunoprecipitations (negative controls) were removed." from the analysis (lines 211-212). We also specify that "Proteins identified but which do not meet these criteria are shown in Table S3." (lines 213-214)

Reviewer #3: (No Response)

Reviewer #4: The title seems to be a bit too general, and the observations should not be generalized to the apicomplexa as evidence was provided only on P. falciparum and T. gondii. It would be nice if the authors touch upon other apicomplexan parasites such as Eimeria spp., Theileria spp., and Cryptosporidium?
Response: We believe that the reference to apicomplexan parasites in the title does not imply that we have shown the complex to be present in ***all*** apicomplexan parasites – just that we have found this complex in some apicomplexan parasites, which we have done. We have now included a statement in the Discussion that reflects how we think our findings in this study extend to the PanKs of other apicomplexan parasites, including those suggested by the reviewer (line 429-430, Figure S13).

Figure 1C: Is it possible to prolong incubation to reach phosphorylation close to 100%?
Response: Yes, this is possible, but since the informative part of the PanK activity curve is the initial part of the time-course, we have not let the reaction run to completion.

Figure 2C: In control, the lysate of the line expressing GFP only did not show any PanK activity (pantothenate phosphorylating) up to 90 min. However, it is thought that in control, the endogenous PanK activity must be detected in the lysate (maybe low activity). Please explain.
Response: We assume that the reviewer means Figure 1c (rather than 2c, which is a western blot). Figure 1c shows activity from GFP-Trap immunoprecipitation samples (not whole cell lysates as the reviewer suggests). In the case of this control, the parasites are expressing un-tagged (free) GFP. Immunoprecipitation of un-tagged GFP from these control parasites should therefore be devoid of PanK activity (as the PanK proteins are not immunoprecipitated).

"PfPanK1 and PfPanK2 are part of an active PanK enzyme complex in P. falciparum parasites". However, one can also argue that PfPanK and 2 make a complex, but only PfPanK1 possesses an enzymological and physiological role. The evidence to claim the statement need to be provided (for instance, make a Pf mutant by removing only one of PfPanK1 and PfPanK2 genes)(if genes not being essential, though), to detect any decrease of PanK activity.
Response: We are of the opinion that the sentence in question does not exclude the possibility that only *Pf*PanK1 possesses active site residues. We have shown in the manuscript that *Pf*PanK1 and *Pf*PanK2 form a complex and that this complex possesses PanK activity – that is the message conveyed by the sentence in question. As for the suggestion of removing either *Pf*PanK1 or *Pf*PanK2 and detecting any decrease in PanK activity – we believe that knocking out either of the isotypes would be lethal to the parasite, as we have observed for the *T. gondii* PanKs (Figure 4). The experiments proposed by the reviewer may therefore not be possible.

5. Fig 3B clearly showed that TgPanK1 and TgPanK2 play independent but cooperative roles for pantothenate phosphorylation. Provide any explanation on any expected specific roles of TgPanK1 and 2 in PanK activity in this parasite?

Response: We believe that the data in Figure 3 (and also the new data in Figure 5) actually show that the two isotypes are *dependent* on each other as both are required to form a functional unit. We propose in the Discussion (lines 422-425) that the two isoforms form a single active site within the heteromeric complex (rather than the two active sites formed by homodimeric PanKs), with key residues for the active site contributed by both isoforms (Fig. S12).
